# SpatialOne: end-to-end analysis of visium data at scale

**DOI:** 10.1093/bioinformatics/btae509

**Published:** 2024-08-17

**Authors:** Mena Kamel, Amrut Sarangi, Pavel Senin, Sergio Villordo, Mathew Sunaal, Het Barot, Seqian Wang, Ana Solbas, Luis Cano, Marion Classe, Ziv Bar-Joseph, Albert Pla Planas

**Affiliations:** Digital R&D, Sanofi, Paris 75017, France; Digital R&D, Sanofi, Paris 75017, France; Digital R&D, Sanofi, Paris 75017, France; Digital R&D, Sanofi, Paris 75017, France; Digital R&D, Sanofi, Paris 75017, France; Digital R&D, Sanofi, Paris 75017, France; Digital R&D, Sanofi, Paris 75017, France; Digital R&D, Sanofi, Paris 75017, France; Precision Medicine & Computational Biology, Sanofi, Vitry-sur-Seine 94400, France; Precision Medicine & Computational Biology, Sanofi, Vitry-sur-Seine 94400, France; Digital R&D, Sanofi, Water Street 450, Cambridge, MA 02141, USA; Digital R&D, Sanofi, Carrer de Rosselló i Porcel 21, Barcelona 08016, Spain

## Abstract

**Motivation:**

Spatial transcriptomics allow to quantify mRNA expression within the spatial context. Nonetheless, in-depth analysis of spatial transcriptomics data remains challenging and difficult to scale due to the number of methods and libraries required for that purpose.

**Results:**

Here we present SpatialOne, an end-to-end pipeline designed to simplify the analysis of 10x Visium data by combining multiple state-of-the-art computational methods to segment, deconvolve, and quantify spatial information; this approach streamlines the analysis of reproducible spatial-data at scale.

**Availability and implementation:**

SpatialOne source code and execution examples are available at https://github.com/Sanofi-Public/spatialone-pipeline, experimental data is available at https://zenodo.org/records/12605154. SpatialOne is distributed as a docker container image.

## 1 Introduction

Spatial transcriptomics (ST) platforms such as 10X Genomics Visium have made it possible to quantify mRNA expression of large numbers of genes within the spatial context of tissues and cells (Moses and Pachter 2022). This technology has facilitated the characterization of expression patterns and regulation in tissues across diverse fields such as developmental biology and neurology (Garcia-Alonso *et al.* 2021, Kasemeier-Kulesa *et al.* 2023). ST also helps in analyzing local features and neighborhoods, contributing to the understanding of rearrangement of cells and structures including the tumor microenvironment, immune responses, cardiac disorders, and neurodegenerative diseases (Zhang *et al.* 2023, Liu *et al.* 2024). Visium combines histological imaging analysis of tissue sections with the detection of the whole transcriptome by attaching them to a glass slide coated with millions of spatially barcoded capture probes. Each Visium slide has four capture areas of 6.5 mm × 6.5 mm. There are a total of 4992 spots per capture area and each spot is 55 μm in diameter with a 100 μm center to center distance between spots (Marx 2021). An important limitation of Visium is that each spot can contain more than one cell, which makes omics and image integration very challenging. A typical Visium analysis workflow consists of preprocessing and normalization, clustering, detection of cell types by signatures, and visualization of gene expression in low dimensions. In addition, recently developed algorithms can be used to perform spot level downstream analysis (Liu *et al.* 2021). Multiple frameworks and tools have been proposed to perform each of these steps. These include Squidpy (Palla *et al.* 2022), Giotto (Dries *et al.* 2021), Cellpose (Pachitariu and Stringer 2022), amongst others. Nonetheless, none of these packages fully cover all processing and analysis tasks, they use different programming languages and frameworks, and have limited interoperability. Their integration requires advanced computational skills, which can pose a significant barrier. This limits the standardization and throughput of analyzing spatial transcriptomics data, making it more challenging to scale the analysis to process multiple experiments quickly and in a reproducible manner. On the other hand, end-to-end pipelines like SpaceMake and ST Tools (Sztanka-Toth *et al.* 2022, Xi *et al.* 2022) do not integrate image analysis in the process, which limits their ability to perform positional and spatial analysis of cells.

Here, we introduce a low-code and automated workflow which integrates state of the art transcriptome and image analysis tools and offers a modular approach framework, allowing users to choose the most appropriated algorithms and parameters for each step. SpatialOne accelerates the analysis of multiple samples and can be useful to expand the adoption of Visium-like technologies, reducing the need for computational expertise. The workflow generates an HTML summary report describing the spatial cell composition of the analyzed tissue, as well as outputs CSV and AnnData files for further analyses. These outputs can be visualized in standard open-source tools like TissUUmaps (Pielawski *et al.* 2023), and facilitate further inquiry into the data using widely adopted scverse packages (Virshup *et al.* 2023).

## 2 Materials and methods

### 2.1 Workflow overview description

SpatialOne requires two inputs: 10x Genomics SpaceRanger software outputs and a study-specific single-cell RNA-seq expression reference dataset. High-resolution hematoxylin and eosin (H&E) stained images are used to identify and pinpoint each cell location by means of deep learning-based image segmentation algorithms. The SpaceRanger-reported Visum spot transcript counts are used to calculate quality control metrics and perform cell deconvolution, identifying the most probable set of cell types responsible for the observed gene expression in tissue sections.

**Figure 1.**
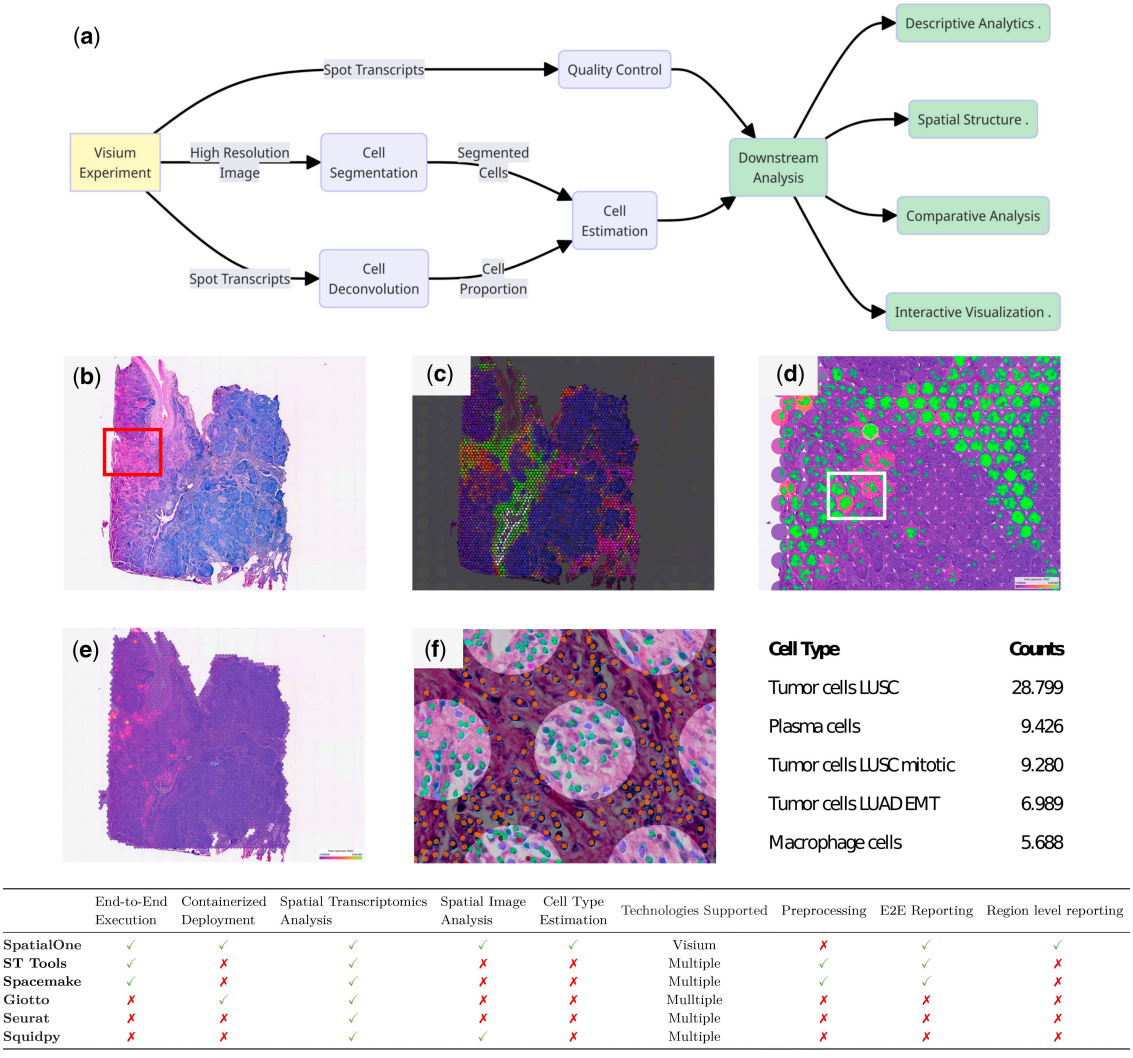
SpatialOne workflow and analysis examples. (a) SpatialOne uses SpaceRanger for expression processing and cell segmentation and cell deconvolution methods to determine cell types. Using the cell location estimation, it generates descriptive analytics, an HTML spatial structure report, compares regions of interest if provided, and displays results in the TissUUmaps interactive viewer. (b) H&E image of a Visium sample corresponding to a human squamous lung cancer slide. Here SpatialOne utilized CellPose for cell segmentation and Cell2Location for cell deconvolution. Single-cell data from the Lung Cancer atlas is used as reference data for deconvolution. (c) Cell type estimation shows the clear distribution of LUSC tumor cells (dark blue), plasma cells (light green), macrophages (red), alveolar cells (pink), fibroblasts (yellow), and ciliated cells (white). (d) Plasma cells (light green cross markers) are present in spots with high IGKC expression. (e) IGKC expression levels. IGKC is used as a marker for plasma cell infiltration in tumors. (f) Cell segmentation and cell type estimation results. Orange dots, in the dark areas, correspond to cells outside Visium spots that cannot be deconvolved. (g) Counts of the Top 5 identified cell types. (Table) Comparison of SpatialOne capabilities versus main existing spatial transcriptomics libraries and analysis pipelines.

By combining visual and expression analysis data (Fig. 1a), SpatialOne estimates the number of cells per spot, enabling various downstream spatial structure analyses, which are compiled into an HTML report for easy review and dissemination. Outputs from SpatialOne, including the HTML report and raw data files, are saved in a user-designated output folder. Results can be visualized in TissUUmaps, an open-source tool for visualizing high-resolution spatial data.

SpatialOne is available as a Docker container, ensuring results reproducibility and ease of installation across computing environments. It uses the Metaflow framework to orchestrate analytical tasks developed in R and Python. Each task is functionally independent, and various implementations of each can be configured—which makes the SpatialOne pipeline modular and promotes flexibility and extensibility by allowing each step to operate independently, processing inputs and generating outputs for subsequent steps. A YAML configuration file allows the user to adjust pipeline parameters, while advanced users can further customize the pipeline by integrating additional processing steps to meet their specific research needs.

### 2.2 Upstream analysis

SpatialOne provides the user with implementations of both default and alternative methods for the steps constituting the spatial data analysis pipeline.

The SpatialOne upstream analysis within the SpatialOne pipeline begins with cell segmentation, leveraging two state-of-the-art methodologies: Cellpose (Pachitariu and Stringer 2022) and HoverNet (Graham *et al.* 2019). Users can tailor these methods to their specific requirements as detailed in the Supplementary Section S1.1. This step produces spatial coordinates for cell nuclei centers, masks for each cell, and morphological features to characterize cells. It also assigns cells to their respective Visium spot.

To estimate the cell types within each Visium spot and their respective proportions, SpatialOne utilizes either Cell2Location or CARD (Kleshchevnikov *et al.* 2022, Ma and Zhou 2022) cell deconvolution algorithm implementations. These cell deconvolution algorithms use the raw transcript counts from the Visium spots to infer spot cell-proportion composition by matching with the closest single-cell RNA expression profiles built by processing the user-provided single-cell reference dataset.

Next, the imaging and deconvolution analyses’ results are combined to predict cell types and their counts within each spot. For each spot, this process takes the number of cells resulting from the cell segmentation and combines it with the deconvolution proportions to estimate the cell count for each cell type. Following, cells are grouped based on their morphological similarities while respecting the counts obtained from cell deconvolution, ensuring cells with similar morphology are labeled with the same type. By integrating the clustering data with the deconvolution-derived cell proportions, we can approximate cell types within a margin of error corresponding to the spot diameter, approximately ±55 µm. This approach yields a view of the tissue cellular landscape at a near single-cell resolution (Fig. 1c–f), enabling spatial structure analysis.

The upstream analysis also includes the generation of quality control metrics for spots in the slide, allowing further inter-sample comparisons (Supplementary Section S1.1.4).

### 2.3 Spatial structure analysis

SpatialOne facilitates a comprehensive quantitative analysis of Visium data, leveraging the near single-cell resolution obtained through the upstream analysis. The combination of proprietary and external methodologies can be divided into four blocks (Supplementary Section S1.2):

- **Descriptive Statistics**: This module summarizes the distribution of cell types across spots while pinpointing the most expressed genes (Fig. 1g).
- **Spatial Analysis**: Using Moran’s I (Behanova *et al.* 2022), this module identifies genes and cells spatial clusters highlighting regions with higher- or lower-than-expected gene expression or cell abundance. It also employs neighborhood enrichment to identify co-occurrence patterns, quantifying how specific cell type pairs tend to associate or disperse within the tissue. This latter analysis is especially important for studies that focus on cell-cell interaction and cell infiltration analysis. Detecting spatial domains can be done in SpatialOne using the Banksy method (Singhal *et al.* 2024) and spatially variable genes are detected using SpatialDE (Svensson *et al.* 2018).
- **Comparative Analysis**: SpatialOne compares gene expression across distinct tissue clusters or regions, providing insights into differential expression patterns. Users can define regions of interest through a custom GeoJSON annotation file or by employing SpaceRanger’s inherent clustering functions.
- **Regional Analysis**: When users supply an annotation file defining areas of interest, SpatialOne produces descriptive and spatial analyses for those regions. It also quantifies cell infiltration within each annotated area by assessing the cell type proportions at varying distances from the region border. This quantification is subjected to a two-sided *Z*-test to determine statistical significance, offering a nuanced view of cellular distribution relative to defined regions.

### 2.4 Outputs and visualization

SpatialOne generates several output files intended for additional data analysis and visualization. The pipeline saves the results of the upstream analysis in both CSV and AnnData formats, encapsulating detailed lists of features that characterize Visium spots and segmented cells. These files are the foundations for generating spatial structure analysis reports, facilitating the reproduction of the analyses.

The spatial structure analysis is documented through a set of HTML reports (Supplementary Material S1.2). The main report presents an overview of the whole sample analysis, while additional per-region of interest reports show corresponding analyses.

For additional interactive visualization, SpatialOne creates a .tmap configuration file for integration with TissUUmaps. This configuration allows the overlay of various layers of upstream results over the high-resolution tissue slide image. Users can interact with and discern between different results layers, including segmentation masks, Visium spots, inferred cell types, gene expression levels, different clustering outputs, and quality control metrics, to facilitate a multi-faceted analysis.

## 3 Results

To showcase the capabilities of SpatialOne, two human lung cancer *formalin-fixed, paraffin-embedded* (FFPE) samples were analyzed. These samples were prepared following the CG000495 protocol (Fig. 1b), sequenced with the 10x Visium CytAssist, and processed using the 10x SpaceRanger software v2. We also tested the pipeline in three adult mouse samples sequenced using 10x Visium samples (one fresh frozen brain tissue section processed using SpaceRanger v2 and two kidney sample processed using the SpaceRanger v1), and 75 internal samples.

Cell segmentation was performed using the CellPose nuclei model, and for cell deconvolution we used Cell2Location. For the human lung cancer samples, single-cell data from the Lung Cancer Atlas (Salcher *et al.*2022) was used as reference-labeled. This dataset was filtered to include only Chromium-generated data. For the mice datasets, the GSE107585 single-cell dataset served as reference for the kidney samples and the one presented in Kleshchevnikov *et al.* (2022) was used for brain. In the human lung cancer datasets, a pathologist annotated regions of interest corresponding to tumors, blood vessels, and alveolar regions. This annotation is used to showcase regional analysis capabilities.

The outcomes of the analyses are presented in an HTML report and visualized through TissUUmaps. This visualization provides a detailed overview of the spatial cellular architecture and gene expression patterns within the tissue samples, as shown in Fig. 1 and Supplementary Section S2.

## 4 Discussion

SpatialOne simplifies spatial studies at scale, ensuring reproducibility and enabling comparative studies when analyzing multiple samples. It is compute platform-agnostic and empowers users without technical expertise. Unlike previous methods (e.g. Squidpy, Giotto, Cellpose, etc.) that often necessitate manual, ad-hoc pipeline development, SpatialOne offers a standardized, and streamlined process (Table in Fig. 1). Its low-code, end-to-end workflow enables researchers to produce robust and reproducible results without the need for advanced computational skills. The modular nature of SpatialOne allows users to customize or extend processing steps.

SpatialOne is built as a Docker container, providing a portable environment that works both on standalone machines and in cloud environments. SpatialOne outputs provide informative and descriptive visualizations that can be interactively queried in TissUUmaps or further analyzed using standard bioinformatics tools.

SpatialOne currently is limited to Visium technology. Thus, any structural analysis involving cellular resolution, such as region infiltration analysis, will have an approximate error of ±55 μm in cell positioning; this corresponds to the Visium spot size. The inclusion of morphological data into the cell type estimation can reduce this error, but its impact is limited as certain cell types cannot be differentiated by their morphology. As a result, we recommend that sub-region analysis performed with SpatialOne should encompass multiple spots. As future work, we plan to expand the SpatialOne to support other ST technologies like VisiumHD, Xenium or CosMx.

SpatialOne is available at https://github.com/Sanofi-Public/spatialone-pipeline, along with the user manual and sample data. The pipeline is maintained by Sanofi Digital, with the intention of continuously integrating various analysis modules and adapting to support emerging technologies, including VisiumHD.

## Supplementary Material

btae509_Supplementary_Data
