# Supplementary material for: SpatialOne: end-to-end analysis of visium data at scale
Source: Bioinformatics. 2024 Aug 17;40(9):btae509. doi: 10.1093/bioinformatics/btae509 (PMC11374018; doi:10.1093/bioinformatics/btae509)
Supplement: btae509_Supplementary_Data [file btae509_supplementary_data.zip › Supplementary_Material.pdf]

*Bioinformatics*, 2024, 1–25
doi: DOI HERE
Supplementary Material
Supplementary Material

# SpatialOne Supplementary Material

## Abstract

This document provides the supplementary material for SpatialOne. Code, installation instructions and examples are publicly available on GitHub at https://github.com/Sanofi-Public/spatialone-pipeline.

## 1. Materials and Methods

This section describes the different methodologies employed at each step of the SpatialOne pipeline. These involve segmenting (Section 1.1.1) the whole slide image to identify cell nuclei, deconvolving (Section 1.1.2) Visium spots to infer their cell phenotype distribution, combining segmentation and deconvolution outcomes (Section 1.1.3) to estimate raw cell counts and their locations, and downstream analysis (Section 1.2) of the results.

### 1.1. Upstream Analysis

#### 1.1.1. Cell Segmentation

In spatial transcriptomics analysis, the initial steps involve analyzing images to understand the tissue sample. Imaging data, in the form of whole slide images, provides the spatial representation of cells in tissue. When combined with gene expression, this added information is critical to understanding cellular function. SpatialOne relies on CellPose (Pachitariu and Stringer, 2022) and Hover-Net (Graham et al., 2019) as methods to perform cell-nuclei segmentation. CellPose aims to generalize well to a wide variety of cell types and tissues, while Hover-Net offers precise segmentations as well as cell-type classification. Both models offer key advantages, especially when considering scale. CellPose and Hover-Net are implemented as two independent modules that the user can select and configure using a configuration file.

In order to integrate CellPose, SpatialOne implements a series of preprocessing and postprocessing steps, which include tissue detection, normalization, patching, and stitching. To work efficiently with larger images, we first split the image into patches and segment the cells in each patch. The segmented patches are then stitched together to form a mask for the entire tissue sample. To deal with overlapping patches and mitigate boundary cell segmentation issues, we store multiple layers of each patch and resolve overlapping cell instances to stitch the patches together and minimize boundary artifacts. We follow the methodology for image patch stitching presented in CellSeg (Lee et al., 2022). CellPose offers both nuclei and cytoplasm segmentation, which the SpatialOne allows users to configure as needed.

Regarding Hover-Net, SpatialOne relies on its original implementation, which is designed to work both with individual tiles or whole slide images. Configurable parameters include save formats, number of cell types of interest if classifying, model mode, and model weights among others. Hover-Net provides three sets of weights: trained with different image datasets, trained with different tissues and with different licensing restrictions. SpatialOne is distributed with the ConSep-derived weights referenced in the original paper. Other Hover-net-specific weights can also be manually inputted for the analysis by the user.

Cells resulting from either nuclei segmentation method are then associated with individual Visium spots. Spot cell counts, together with cell deconvolution outputs, are used as input to the cell type estimation process.

#### 1.1.2. Cell Deconvolution

Spatial spot deconvolution aims to decipher complex gene expression patterns observed within Visium spots by identifying the most likely combination of cell types that could produce such patterns. The search for this combination is carried out by means of a tissue-specific single-cell RNA-seq reference dataset (i.e., single-cell atlas, such as in (Salcher et al., 2022)) provided by the user, which is a collection of observed gene expressions for each cell type within the studied tissue. Typically, the deconvolution process builds upon advanced computational techniques, including machine learning and statistical models. The literature offers other deconvolution algorithms that do not rely on reference data, such approaches offer more flexibility but are less accurate Li et al. (2022, 2023), thus they are not considered in SpatialOne.

SpatialOne users can choose between two reference-labelled deconvolution methods: CARD (Ma and Zhou, 2022) and Cell2location (Kleshchevnikov et al., 2022), which can be enabled in the configuration file. These two methods were selected following recommendations by Li et al. (2023). Although both predict tissue cellular composition through spatial transcriptomics data and reference RNA-seq dataset analysis, they employ distinct approaches. CARD focuses on assigning cell types to spatial transcriptomics spots based

© The Author 2024. Published by Oxford University Press. All rights reserved. For permissions, please e-mail: journals.permissions@oup.com 

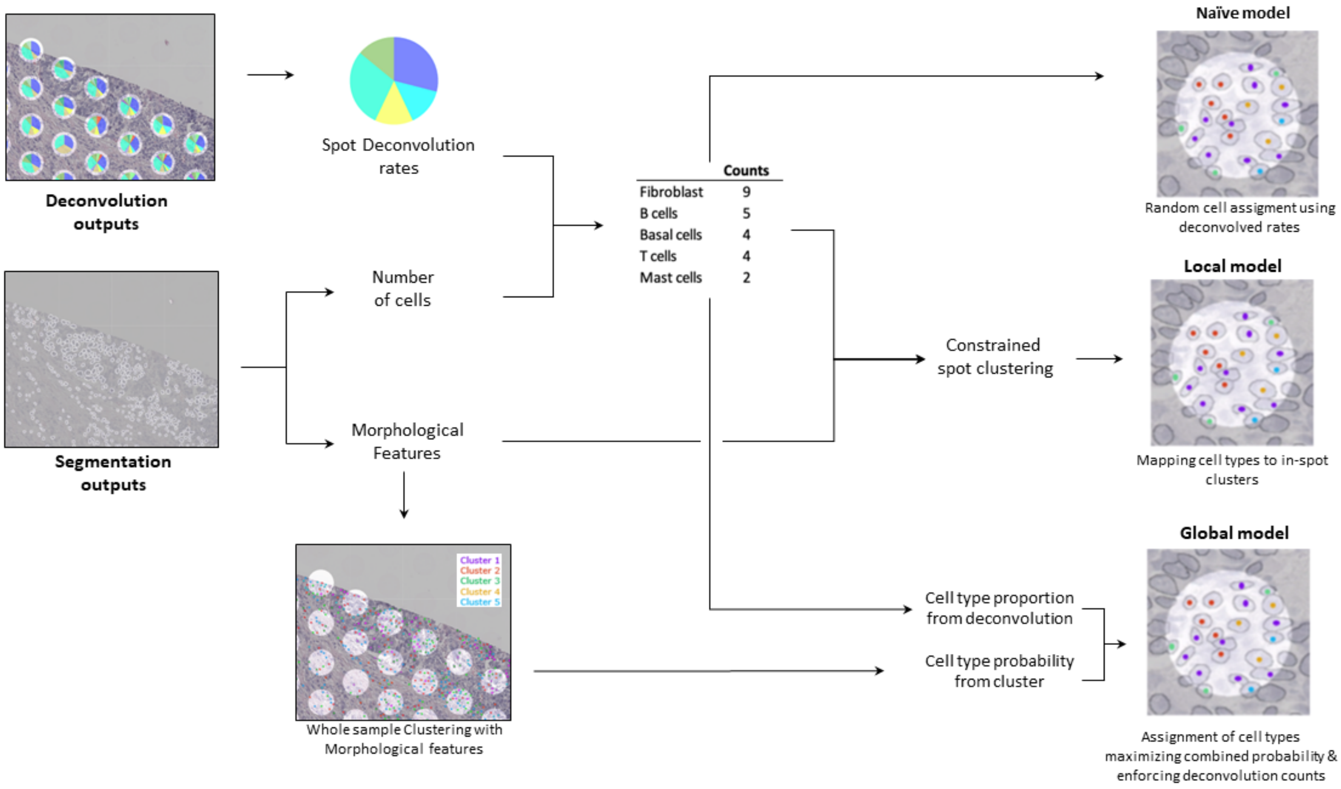


Fig. 1: SpatialOne Cell Type Estimation methods

on relative proportions, using reference scRNA-seq data. In contrast, Cell2location provides a more detailed quantification of cell type abundances within each spot, employing a Bayesian framework to integrate spatial and expression data. The choice between CARD and Cell2location would depend on the specific requirements of the study, including the need for relative versus absolute quantification of cell types and the level of detail required in understanding the spatial organization of the tissue.

After determining the estimated cell proportions for each spot, SpatialOne integrates this data with the results from cell segmentation to calculate the counts of each cell type per spot. Converting the number of segmented cells within a spot (a single integer number) into a set of cell type counts satisfying the predicted cell type proportions (fractions) results in a precision loss, which we measure as earth mover distance (EMD) error (Rubner et al., 1998) and minimize via a greedy iterative approach. Thus, to compute the counts, we assign cells one by one to the predicted proportion bins, choosing the bin that minimizes the accumulated EMD value at each step. Specifically, the deconvolution algorithm output provides the reference distribution $D_f$ of size $K$ ($K$ cell types) for a Visium spot. The $D_f$ values are floats. Via a greedy iterative algorithm, we compose the optimal integer-valued distribution $D_i$ of size $K$ that assigns $N$ cells (the count of cells identified by the cell segmentation algorithm) into $K$ bins while minimizing $Diff(D_f, norm(D_i))$. On each of the $N$ steps, the greedy algorithm assigns a cell to the bin of $D_i$ that minimizes the difference value. As $D_i$ is a vector of integer cell counts of size $K$, to compute the difference, we normalize $D_i$ by dividing the vector of counts per cell type by the total count of assigned cells so far.

### 1.1.3. Cell-Type Estimation

The 10x Visium technology resolution is limited to the spot size of 55 µm, which is insufficient for single-cell resolution. To increase the resolution for the downstream analysis, we developed three cell type estimation methods that use outputs from previous steps to approximate cell-type labels for each segmented cell (Figure 1). The three proposed methods use different estimation approaches with varying levels of complexity to assign spot cell labels while enforcing deconvolution proportions. These methods range from a naive model that performs random assignment, analogous to Tangram's (Biancalani et al., 2021) cell assignment methodology, to increasingly sophisticated local and global algorithms that integrate the cell counts inferred during the deconvolution step with the morphological features of the image.

#### Naïve Model

The naïve method propposed by Biancalani et al. (2021) assigns cell types randomly while preserving the cell deconvolution proportions of each spot. As shown in the Supplementary Parameters Table (Supplementary Table 2), the naïve model can be parameterized by specifying a random seed, allowing users to modify its default value to generate different random assignments.

**Local Model**

The local method uses a size-constrained clustering (Ganganath et al., 2014) algorithm based on deterministic annealing (DA) to group cells within each spot based on morphometric features. It relies on the `size-constrained-clustering` package implementation. The size-constrained DA algorithm takes a vector specifying the size for each cluster as input. The local method uses the cell type raw counts obtained from deconvolution to determine the expected size of each cluster. This allows assigning the same cell type to morphologically similar cells within a spot while maintaining deconvolution proportions. Thereby, SpatialOne first extracts morphological features from segmentation masks using the `regionprops` function from `scikit-image` (van der Walt et al., 2014). This function quantifies various image properties including the area, centroid, intensity and bounding box dimensions for each segmented nucleus. Following feature extraction, the pipeline performs data preprocessing in two steps: (i) feature scaling and (ii) dimensionality reduction using principal component analysis (PCA), resulting in the selection of the first 10 principal components. These principal components serve as the input of the clustering together with the expected cluster sizes derived from cell deconvolution. In cases where multiple cell types have the same cell count, the cell type estimation is resolved by randomly selecting one of the cell labels for the cluster.

As shown in the supplementary Parameters Table (Supplementary Table 2), the local model can be parameterized by passing a random seed to the clustering algorithm. In parallel, the number of clusters is equal to the number of unique cell types in the spot and their size with the number of cells of each type.

**Global Model**

The global method refines the cell type estimation process by leveraging whole-image information. It applies k-means clustering to group cells across the entire slide by their morphological features using the `scikit-learn` library (Pedregosa et al., 2011). Morphometric features are computed in the same fashion as in the local model (Figure 1). For each identified cluster, a score is computed to represent how likely a given cell from that cluster is to belong to a particular cell type. To build such a score, it utilizes the deconvolution estimated counts from corresponding spots. For each identified cluster, the method computes the weighted average of cell proportions across all spots that contain at least one cell from the cluster. The weight for each spot's contribution is determined by the number of cells from the cluster within that spot. The calculated average is then normalized to ensure it sums to one. Subsequently, for each spot, the model looks for the combination of cell labels that maximizes the aggregated score of each cell

Since the problem of generating all the potential cell label combinations in a spot has a complexity of $\mathcal{O}(n!)$, being $n$ the number of cells in this spot, this method uses a metaheuristic approach to search for a pseudo-optimal assignment. Thus, SpatialOne applies the simulated annealing algorithm implemented in the `frigidum` library (Hendriksation, 2020) to find an optimal estimation by minimizing a fitness function that is defined as

$$\text{Fitness} = 1 - \frac{\sum_{i=1}^{n} P_i^c}{n}, \tag{1}$$

where $n$ is the number of cells of a given spot and $P_i^c$ is the score that the $i^{\text{th}}$ cell belongs to the assigned cell type. While simulated annealing cannot guarantee the globally optimal solution, it efficiently finds high-quality solutions (Dekkers and Aarts, 1991), making it suitable for this application.

As shown in the supplementary Parameters Table (Supplementary Table 2), the global method can also be parameterized by specifying the number of clusters, the batch size and the random state for the morphological clusters. Although we provide a default value of 20, which has proven to perform well when the number of cell types is not excessively high, as a rule of thumb, we recommend selecting a number of clusters higher than the number of expected cell types.

Due to its higher complexity, the global allocation method is significantly slower than the naive and global models.

**Limitations of the Cell Type Estimation algorithms**

Visium's technology limitations can impact the accuracy of cell abundance estimation, especially for spot-based methods that might be involve partially captured cells. This issue is crucial during the cell deconvolution step, where distorted cell proportions can affect results. Similarly, we acknowledge that cell morphology alone cannot be used to accurately identify certain cell types especially when differentiating between subtypes. The cell type estimation method does not aim to impute gene expression per cell as other methods claim Vahid et al. (2023), given Visium technology limitations this can lead to unrealistic scenarios. Instead, we provide realistic cell counts and distributions across tissue spots, enabling structural analysis based on cell positioning. Considering the potential error of ± 55 µm in cell positioning is due to the Visium spot size, we recommend using cell type estimation to analyze broader areas and not focus on specific spot results. to help users understand labelling accuracy and limitations, SpatialOne includes uncertainty metrics about the cell positioning estimates, such as relative entropy of the spot, the silhouette scores of clustering, as well as a confidence score that combines both of these measurements by the following formula:

$$C_s m = (1 - \text{relative\_entropy(s)}) \cdot \min\left(1, \text{silhouette\_coefficient(m)}\right) \tag{2}$$

where $s$ corresponds to the spot a cell belongs and $m$ to its morphological cluster.

To further clarify our cell type estimation methodology, we performed additional analyses using a Visium-like synthetic dataset based on the Lizard dataset (Graham et al., 2021). We evaluated performance in ideal and realistic scenarios, considering in-spot cell heterogeneity. The analysis quantified the impact of noise on cell assignment accuracy, showing that SpatialOne is robust to low levels of noise, although significant cell loss can lead to errors - highlighting the importance of a robust cell segmentation methodology. The tests also show how cell type heterogeneity in the spot condition the reliability of the cell estimation.

The Lizard dataset is a large instance segmentation and classification dataset comprising histology image regions and corresponding instance mask labels. This dataset was built using Colon Cancer images collected from five different previous datasets (DigestPath, GlaS, PanNuke, CRAG, CoNSeP) at 20× objective magnification, with annotations for six types of nuclei (Epithelial cell, Connective tissue cells, Lymphocytes, Plasma cells, Neutrophils, Eosinophils). These annotations were taken as the ground truth labels to be compared with the predictions of our algorithms. We selected accuracy as a metric to evaluate the performance of the cell type estimation methods. To generate the Visium-like synthetic dataset, we simulated artificial spots on top of the histology images, arranging them as non-overlapping circumferences of 55 $\mu$m of diameter. We assigned cells to their corresponding spot based on the coordinates of their segmentation mask centroids. The generated spot level dataset contained 18,110 spots with a total of 241,565 labeled nuclei.

To simulate the gene expression of each spot we took the ground truth cell type labels from Lizard and counted the number of cells of each type for each spot. We then generated gene expression profiles following the methodology described by Ma and Zhou (2022), and retrieving the single cell expression profiles from Drokhlyansky et al. (2020).

We tested the cell type estimation methods in two scenarios:

- To test our image analysis methods we used the ground truth cell type labels for each spot and computed the number of cells of each cell type in each spot, simulating the ideal scenario where cell deconvolution is perfect.
- In order to illustrate deconvolution and segmentation impact, we ran cell deconvolution using cell2location and obtained the cell segmentation masks from Hovernet.

As an output of each test, we obtained the estimation of cell types to segmented cells with the different implementations of the algorithm. Then, we computed the accuracy individually for each spot using ground truth labels and we took the mean to generate a single metric. Results are shown in Table 1.

**Table 1.** Cell type estimation performance on Lizard dataset.

| Input proportions | Cell mask inputs | Cell type estimation algorithm | Accuracy |
|---|---|---|---|
| Ground truth | Ground truth | Global | 0.772 |
| Ground truth | Ground truth | Local | 0.685 |
| Ground truth | Ground truth | Naïve / Tangram | 0.666 |
| Cell2Location | Hovernet | Global | 0.551 |
| Cell2Location | Hovernet | Local | 0.524 |
| Cell2Location | Hovernet | Naïve / Tangram | 0.515 |

To assess the performance of the cell estimation method in spots of different complexity, we selected groups of spots from different datasets (DigestPath, GlaS, PanNuke, CRAG, CoNSeP) that had different cell type heterogeneity. We then calculated the estimation accuracy based on the number of cell types present at each spot, shown in Table 2.

**Table 2.** Cell type estimation algorithm performance.

| Cell type estimation algorithm | Total Accuracy | 1 type | 2 types | 3 types | 4 types | 5 types | 6 types |
|---|---|---|---|---|---|---|---|
| Global | 0.772 | 1.000 | 0.838 | 0.672 | 0.526 | 0.443 | 0.401 |
| Local | 0.685 | 1.000 | 0.748 | 0.513 | 0.377 | 0.344 | 0.326 |
| Naïve / Tangram | 0.666 | 1.000 | 0.677 | 0.502 | 0.374 | 0.312 | 0.287 |

Results show that incorporating morphological information into the cell type estimation improves the baseline naïve approach proposed by Biancalani et al. (2021).

#### 1.1.4. Quality Control

RNA sequencing methodology behind 10x Visium is powerful enough to cover whole human transcriptome from fresh-frozen or even formalin-fixed paraffin-embedded (FFPE) samples (Liu et al., 2022). However, raw RNA-seq data may present quality issues arising from sample collection, preservation, processing or sequencing, which can significantly distort analytical results and lead to erroneous conclusions (Zhou et al., 2018). In this context, some standard quality control (QC) metrics are commonly used to ensure that data quality is sufficient for downstream analysis (Luecken and Theis, 2019; Avila Cobos et al., 2020). For that, SpatialOne computes a set of standard QC metrics (Table 3) derived from the gene count matrix that can be used to evaluate the quality of experiments and remove low-quality spots or genes as is typically done in the literature ( Zhang et al. (2023); Liu et al. (2022); Du et al. (2023); Li et al. (2022))

As with any other step in the SpatialOne pipeline, QC metrics are automatically computed during the pipeline execution according to the setup provided in the configuration file.

### 1.2. Downstream Analysis: Spatial Structure Report

SpatialOne's reporting module generates an HTML report with tables and interactive Plotly figures, providing a high-level view of the cellular and genetic makeup of the tissue under study. The report incorporates several spatial statistical tests such as neighborhood

| Metric | Level | Description |
|---|---|---|
| $n_{\text{Genes}}$ | Spot | Number of genes detected at each spot. |
| $n_{\text{Genes\_prop}}$ | Spot | Proportion of genes detected out of the total number of genes analyzed. |
| $n_{\text{Counts}}$ | Spot | Total amount of mRNA detected by each spot. |
| $n_{\text{Counts\_prop}}$ | Spot | Proportion of detected mRNA out of the total detected for each spot. |
| $CountByGene$ | Spot | Ratio between $n_{\text{Counts}}$ and $n_{\text{Genes}}$ metrics. |
| $GeneSaturation$ | Spot | CountByGene derived metric $[(1 - n_{\text{Genes}}/n_{\text{Counts}}) * (n_{\text{Counts\_prop}})]$. |
| $mitochondrial\_genes\_n$ | Spot | Number of distinct mitochondrial genes detected at each spot. |
| $mitochondrial\_genes\_cts$ | Spot | Total counts detected for mitochondrial genes at each spot. |
| $num\_contained\_cells$ | Spot | Number of segmented cells at each spot. |
| $n_{\text{Spots}}$ | Gene | Number of covered spots by each gene. |
| $n_{\text{Spots\_prop}}$ | Gene | Proportion of covered spots out of the total number of spots. |
| $n_{\text{Counts}}$ | Gene | Total amount of mRNA detected for each gene. |
| $n_{\text{Counts\_prop}}$ | Gene | Proportion of detected mRNA from total for each gene. |
| $CountsBySpot$ | Gene | Ratio between nCounts and nSpots metrics. |
| $SpotSaturation$ | Gene | CountBySpot derived metric $[(1 - n_{\text{Spots}}/n_{\text{Counts}}) * (n_{\text{Counts\_prop}})]$. |
| $mitochondrial\_genes$ | Gene | Boolean indicating if gene is a Mitochondrial gene. |
| $detected\_probes$ | Whole experiment | Total number of times genes were detected over the whole experiment. |
| $library\_size$ | Whole experiment | Total counts of mRNA detected by the experiment. |
| $CountByProbes$ | Whole experiment | Ratio between library size and detected probes. |

**Table 3.** QC metrics computed within the SpatialOne pipeline

| | | Whole Tissue Report | Region Report |
|---|---|---|---|
| **Descriptive Statistics** | Cell summary statistics | ✓ | ✓ |
| | Average gene expression of top $N$ genes | ✓ | ✓ |
| **Spatial Analysis** | Neighbourhood enrichment analysis | ✓ | ✓ |
| | Co-occurrence analysis | ✓ | ✓ |
| | Moran's I analysis | ✓ | ✓ |
| | SpatialDE | ✓ | ✗ |
| **Comparative Analysis** | Infiltration analysis | ✗ | ✓ |
| | Differential gene expression | ✓ | ✗ |

**Table 4.** Comparison of whole-tissue and region report content.

enrichment analysis (Schapiro et al., 2017), Moran's I analysis (Moran, 1950), and co-occurrence analysis (Tosti et al., 2021), leveraging Squidpy's implementation (Palla et al., 2022a). In addition, SpatialOne enables the same level of insight on isolated tissue regions specified by GeoJSON or CSV annotation files. For each region, the reporting module generates an individual region-specific report describing the subset of cells, Visium spots, and genes. In addition, the proportion of cell types is computed as a function of distance to the region boundary, facilitating the drawing of conclusions on the cell types that infiltrate the region of interest. Finally, the reporting module computes the differential gene expression across all the defined tissue regions and clusters, providing an indication of the gene's regulation pattern. Table 4 provides an overview of the different analyses available through the whole-tissue and region-level reports.

#### 1.2.1. Descriptive Statistics

Cell Summary Statistics

SpatialOne summarizes the types of cells found in the tissue and how they are distributed within Visium spots. Figure 2 shows a sample cell summary statistics table generated by SpatialOne. Figure 3 shows the plot for visualizing the number of cells within the tissue, while Figure 3b represents the corresponding distribution of cell types within a spot.

Average Gene Expression

This section summarizes the top $N$ genes with the highest expression levels, where $N$ is set in the report configuration file. This summary is created by calculating the average gene expression across all Visium spots in the specified tissue or region of interest. Figure 4 shows a plot generated by SpatialOne to visualize the average gene expression for the top $N$ genes in the specified spots.

| cell_type | cell_count | avg_spot | median_spot | max_spot | min_spot | std_spot | p90_spot | p10_spot |
|---|---|---|---|---|---|---|---|---|
| macrophages | 854 | 2.12 | 1 | 12 | 0 | 2.18 | 5 | 0 |
| epithelial cells | 512 | 1.27 | 0 | 24 | 0 | 3.95 | 3 | 0 |
| keratinocytes | 432 | 1.07 | 0 | 22 | 0 | 3.18 | 2.9 | 0 |
| cd4+ t-cells | 357 | 0.89 | 0 | 9 | 0 | 1.27 | 3 | 0 |
| cd8+ t-cells | 297 | 0.74 | 0 | 6 | 0 | 1.05 | 2 | 0 |
| endothelial cells | 217 | 0.54 | 0 | 5 | 0 | 0.73 | 1 | 0 |
| fibroblasts | 199 | 0.5 | 0 | 6 | 0 | 0.66 | 1 | 0 |
| b-cells | 140 | 0.35 | 0 | 3 | 0 | 0.57 | 1 | 0 |
| dc | 94 | 0.23 | 0 | 2 | 0 | 0.43 | 1 | 0 |
| melanocytes | 94 | 0.23 | 0 | 3 | 0 | 0.6 | 1 | 0 |
| hsc | 57 | 0.14 | 0 | 2 | 0 | 0.4 | 1 | 0 |
| adipocytes | 53 | 0.13 | 0 | 2 | 0 | 0.37 | 1 | 0 |

Fig. 2: Sample cell summary statistics table. *cell_count* describes the total number of cells within the tissue. *avg_spot*, *median_spot*, *max_spot*, *min_spot*, *std_spot*, *p90_spot*, and *p10_spot* describe the mean, median, max, min, standard deviation, 90$^{\text{th}}$ percentile, and 10$^{\text{th}}$ percentile of the cell type within all the Visium spots under study, respectively.

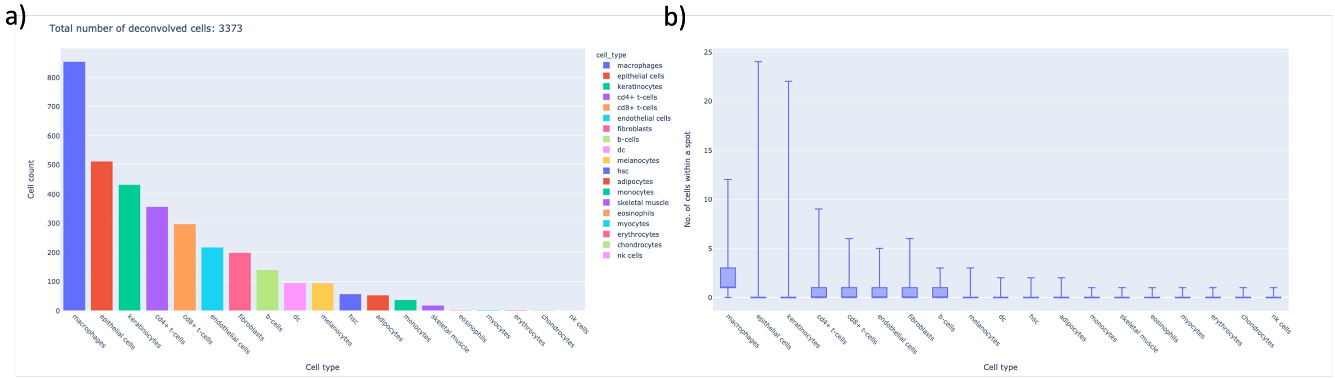


Fig. 3: Cell counts plots. a) Total number of cells available in the tissue or region of interest. b) Distribution of cell types within all the tissue or region spots.

### 1.2.2. Spatial Analysis

#### Neighbourhood Enrichment Analysis

This test identifies cell pairs exhibiting either a higher tendency to co-occur or to disperse more frequently than expected by random chance. This requires constructing a neighborhood graph, connecting cells to their surrounding neighbours. Several techniques can be used for constructing the neighborhood graph (Figure 5), including Delaunay Triangulation or radial distance (Palla et al., 2022b).

Based on cell connectivity, if two distinct cell types are found in close proximity of each other more often than in a random permutation of objects, the test assigns a high Z-score, indicating that the cell types are enriched. Conversely, if the two cell types are far apart, the test assigns a negative Z-score, indicating that they are depleted. Figure 6 shows the plots available for visualizing neighborhood enrichment results.

#### Co-Occurrence Analysis

The co-occurrence score measures the co-occurrence of two different cell types in the tissue (Tosti et al., 2021). For a cell type of interest, $cell_{\text{target}}$, this test measures the probability of observing any other cell type, $cell_{\text{other}}$, within a given radial distance. Co-occurrence score is given by Equation 3:

$$\frac{p(cell_{\text{other}}|cell_{\text{target}})}{p(cell_{\text{other}})}, \tag{3}$$

where $p(cell_{\text{other}}|cell_{\text{target}})$ is the probability of observing $cell_{\text{other}}$, conditioned on the presence of $cell_{\text{target}}$. $p(cell_{\text{other}})$ is the probability of observing $cell_{\text{other}}$ within the radius of interest (Palla et al., 2022b). The score is computed at increasing radii around each cell in the tissue and the results are plotted in a line plot showing co-occurrence score as a function of distance. The radius is defined as the distance

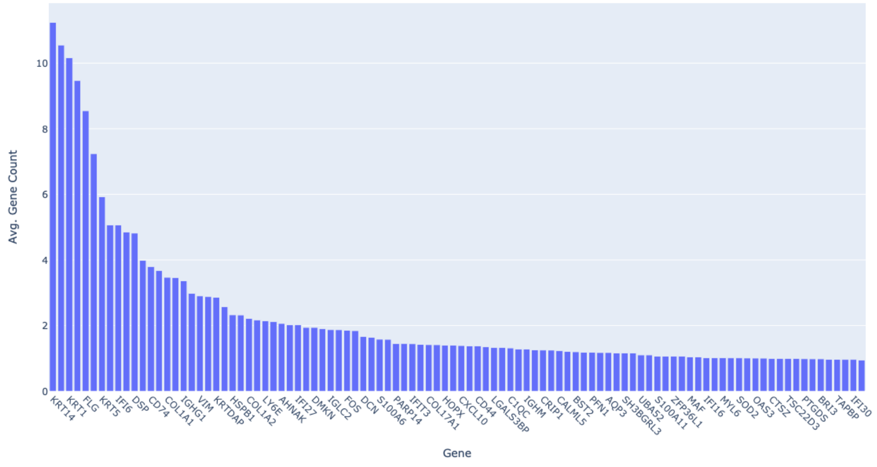


Fig. 4: Average gene expression of the top $N$ genes within the tissue.

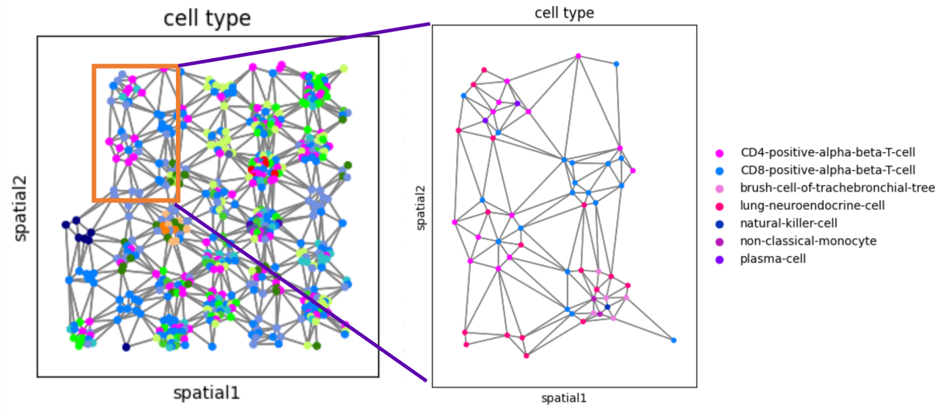


Fig. 5: Cell neighborhood construction using Delaunay triangulation.

between the centers of two consecutive spots ($\sim 65\,\mu$m). Figure 7 shows an example plot for visualizing the results from co-occurrence analysis.

### Moran's I Analysis

Moran's I (Moran, 1950) measures spatial autocorrelation by measuring correlation in a signal among nearby locations in the tissue. This metric is used to measure whether genes and/or cells are clustered, dispersed, or are randomly distributed in the tissue or region being studied. Moran's I can also be used to identify spatially expressed genes. Computing the metric requires building a neighborhood graph for cells and Visium spots under study (Palla et al., 2022b), as mentioned in the "Neighbourhood Enrichment Analysis" section (see Figure 5). For Visium spots, $n$ dictates the radial distance to consider when defining the neighboring spots. Figure 8 shows a typical neighborhood graph of Visium spots. Figure 9 shows Moran's I score for the top cells and genes present in the tissue.

### Spatial Domain Identification and Spatially Expressed Genes

Spatial analysis typically includes the identification of spatial domains (sub-regions of the tissue that share similar gene expression patterns) and spatially expressed genes (groups of genes that follow different expression patterns in different tissue sub-regions). Despite those can be carried out by statistical methods like Moran's I or clustering algorithm, ST state-of-the-art provides more advanced techniques to address these challenges. To this end, SpatialOne incorporates Banksy (Singhal et al., 2024) and SpatialDE (Svensson et al., 2018) outputs into the spatial structure analyis report.

Banksy allows for detecting spatial domains or regions that have unique gene expression profiles. This helps with understanding the organization of tissues and how specific regions may contribute to disease progression. The method involves creating clusters based on gene expression patterns, which are then refined using both gene expression data and spatial coordinates of the spots to assign probabilities of each spatial location. After ensuring that the detected domains are spatially contiguous, Markov Chain Monte Carlo sampling is used to iteratively estimate the posterior distribution of the domain assignments.

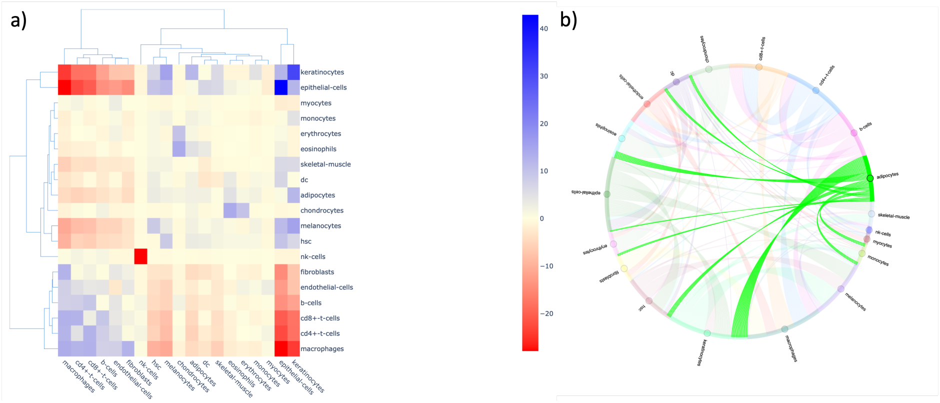


Fig. 6: Neighborhood enrichment analysis plots. a) Clustergram for visualizing Z-scores. Blue indicates enrichment between two cell types. Red indicates depletion between two cell types. b) Chord plot for visualizing cell enrichment scores. Chord width indicates strong enrichment between the cell types.

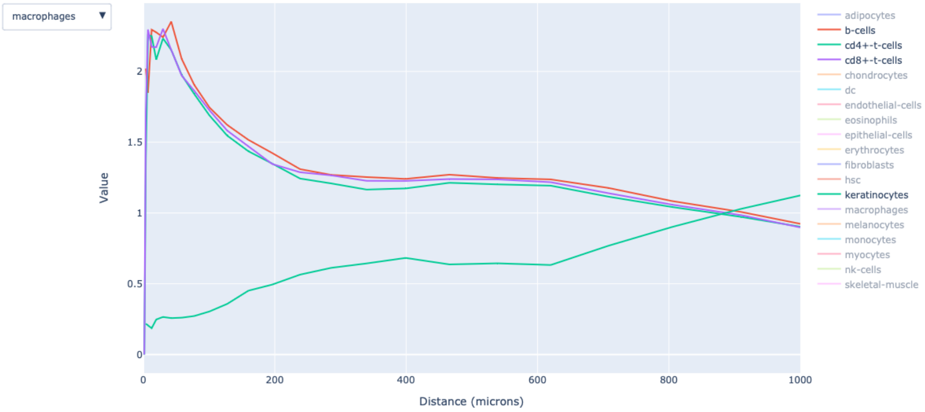


Fig. 7: Visualizing co-occurrence of B-cells, CD4+ T cells, CD8+ T cells, and Keratinocytes ($cell_{\text{other}}$) as a function of distance away from macrophages ($cell_{\text{target}}$).

SpatialDE is a method that identifies genes with spatially variable expression patterns. It builds on Gaussian process regression, a family of models popular in geostatistics. SpatialDE first normalizes the gene expression counts from all the Visium spots. Then, for each gene, it decomposes the expression variance into spatial and non-spatial components. The fraction of variance explained by spatial variation (FSV) is computed by taking the ratio of the variance explained by those two components. Statistical significance of the spatially variable genes is computed by comparing the full model, which includes both spatial and non-spatial components, to a model without the spatial variance component. Figure 10 shows the highest spatially variable genes along with their gene distribution patterns.

### 1.2.3. Comparative Analysis

SpatialOne incorporates the functionality the ability to compare user-defined regions in two formats:

- **.geojson:** Format used to label tissue regions or landmarks. SpatialOne runs a background process to extract the Visium spots and cells located within each user-annotated region for downstream analysis.
- **.csv:** Formatused to label Visium spot annotations either computationally (e.g. using a clustering algorithm), or manually by an expert. The file requires a minimum of two columns: “barcode” and “visium_cluster”, describing the Visium spot barcode and the corresponding spot annotation, respectively.

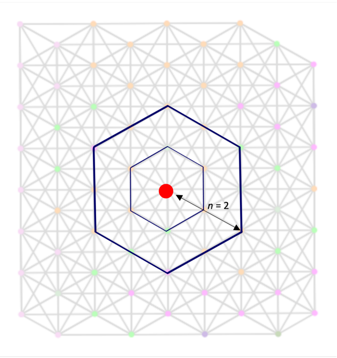


Fig. 8: Visium spot neighborhood graph used for computing Moran's I. For any given Visium spot (shown in red), $n_{\text{rings}} = 2$ defines a spot neighborhood two radial distances wide.

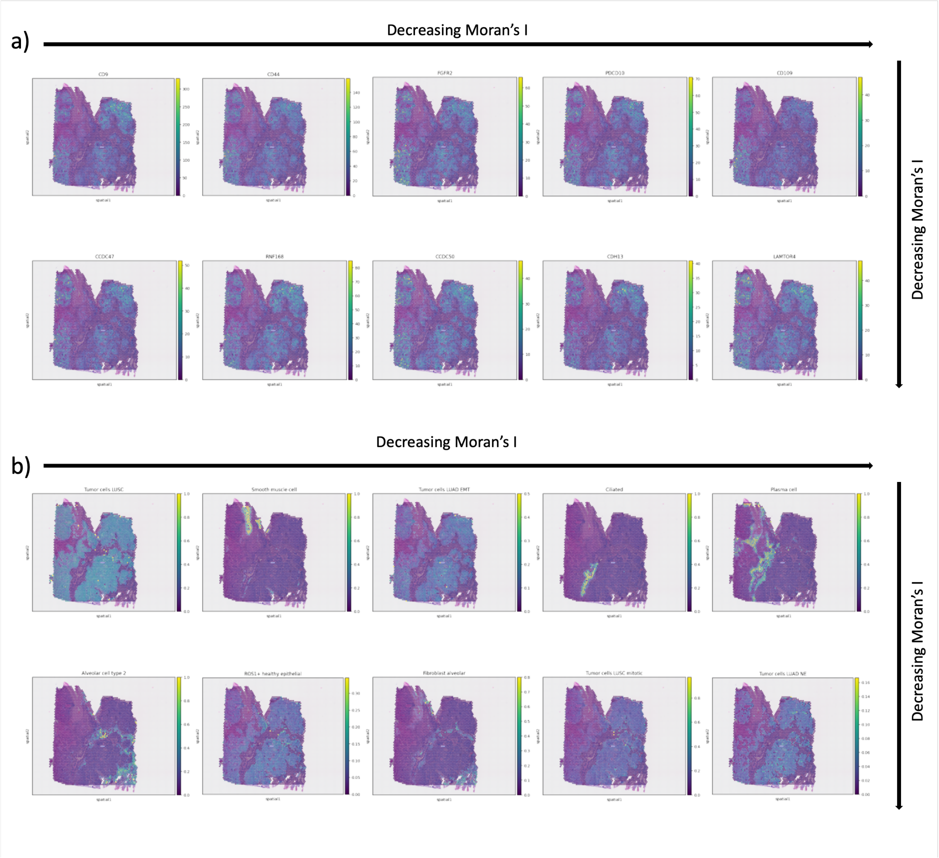


Fig. 9: Visualizing Moran's I as a heat map. a) Plotting Moran's I for the top 10 genes with the highest Moran's index. Those genes have the highest degree of spatial auto-correlation. b) Plotting Moran's index for the top 10 cell types with the highest degree of spatial autocorrelation.

Infiltration Analysis

For each user-provided region, SpatialOne's reporting module computes cell proportions as a function of distance to the region boundary. The process starts by creating distance intervals of study by eroding and dilating the region of interest, as shown in Figure 11a. At each distance interval, cell type proportions are computed, providing a cell type distribution as a function of distance to the region boundary.

A two-sided Z-test of proportions is used to assess if the proportion of cells inside a region, $p(\text{inside})$ is equal to the proportion of cells outside a region, $p(\text{outside})$. Cell types with a significantly different proportion across the region boundary are marked for further study by the user. To visualize the cell type differences across the boundary, two methods are provided through SpatialOne region reports: cell type abundance per distance interval (Figure 11b) and cell type proportion as a function of distance (Figure 12).

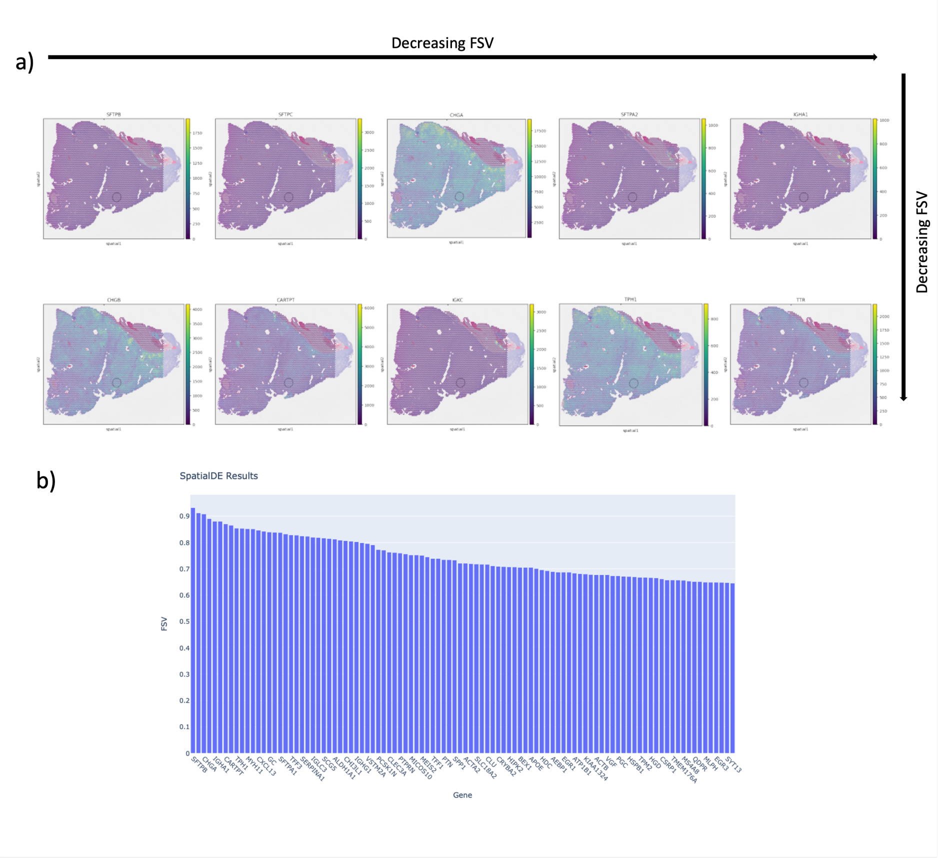


Fig. 10: Visualizing genes with significant spatial variance identified by SpatialDE. (a) Spatial distribution of gene expression for the top 10 genes with the highest fraction of spatial variance (FSV). (b) Alternative plot showing the top 100 genes with the highest FSV in the sample.

### Differential Gene Expression

This test compares the gene regulation pattern between two regions of interest. By default, SpatialOne compares all combinations of regions provided by the user. The results are visualized in a volcano plot with colors signifying regulation patterns as up-regulated, down-regulated, or not significant (Figure 13).

Given two regions, $region_{\mathrm{A}}$ and $region_{\mathrm{B}}$, the algorithm gets the list of genes mutually expressed in both regions. For each gene, the fold change $fc$ is then computed according to the following equation:

$$fc = \frac{\mu_{\mathrm{A}}}{\mu_{\mathrm{B}}}, \tag{4}$$

where $\mu_{\mathrm{A}}$ and $\mu_{\mathrm{B}}$ are the gene's mean expression level in $region_{\mathrm{A}}$ and $region_{\mathrm{B}}$, respectively.

To test if the two regions have a significantly different gene expression mean, T-test and Mann-Whitney U tests are run followed by Benjamini-Hochberg correction (Benjamini and Hochberg, 1995) to decrease the false discovery rate. A gene is considered significantly different between $region_{\mathrm{A}}$ and $region_{\mathrm{B}}$ if the corrected $p$-values from both T-test and Mann-Whitney U test are less than the default significance value (0.005).

Fold change is used to determine the directionality of the gene regulation pattern. A gene is up-regulated in $region_{\mathrm{A}}$ with regards to $region_{\mathrm{B}}$ if $fc$ is greater than the threshold value, $fc_{\mathrm{threshold}}$ (default value is 3). Conversely, a gene is down-regulated in $region_{\mathrm{A}}$ with regards to $region_{\mathrm{B}}$ if $fc$ is less than $1/fc_{\mathrm{threshold}}$.

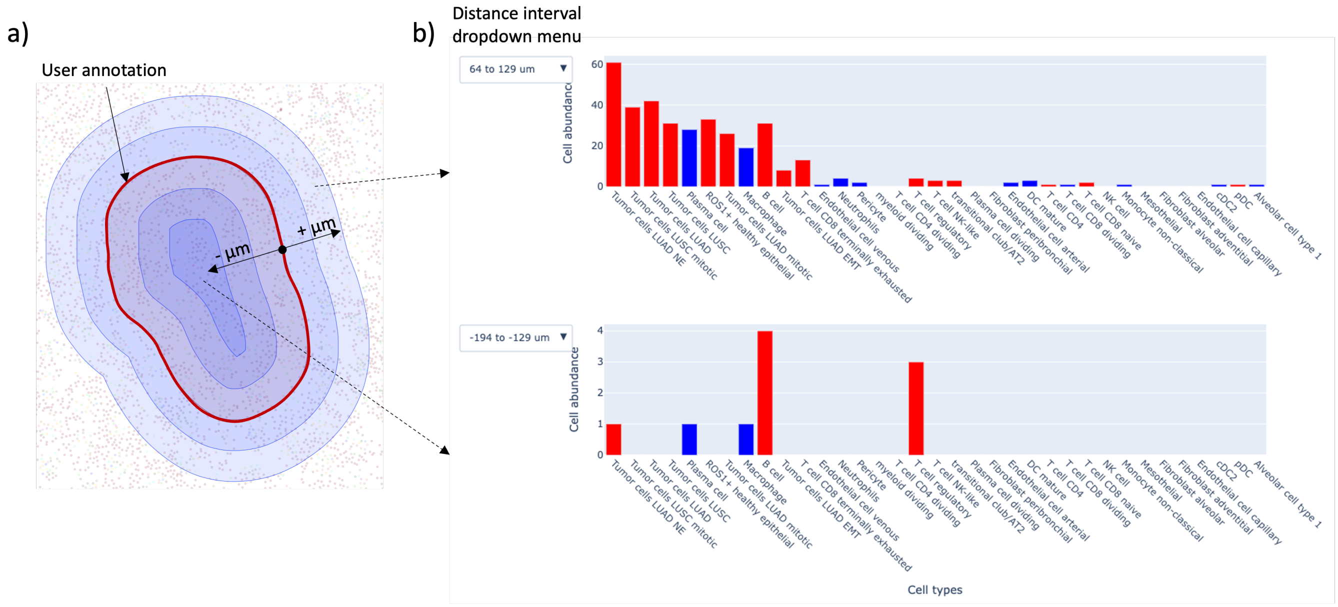


Fig. 11: a) Distance intervals of study for a given user annotation. Positive distances are distances away from the boundary, while negative distances are inside the boundary. b)Visualizing cell type abundance per distance interval of study. SpatialOne region reports provide a drop-down menu to select the distance interval to study in microns. The dotted arrows relate each pannel with its corresponding expanded area. Cell types with significantly different proportions inside and outside the region are depicted in red.

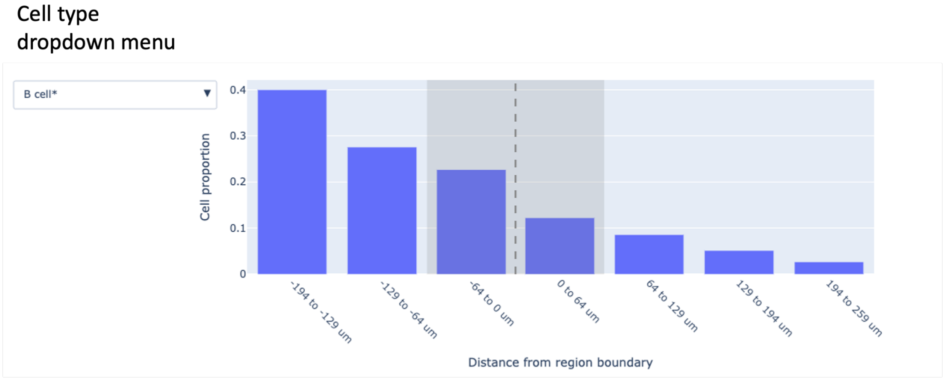


Fig. 12: Visualizing cell type proportion as a function of distance to the region boundary. SpatialOne region reports provide a drop-down menu for selecting the cell type to study. $y$-axis shows the cell type proportion while the $x$-axis shows the distance to the region boundary. The grey area represents intervals in the immediate proximity to the region boundary. These intervals are omitted from the Z-score computation to avoid errors due to inaccurate cell type estimation. Cell types with significantly different proportions inside and outside the region are labeled with an asterisk in the drop-down menu.

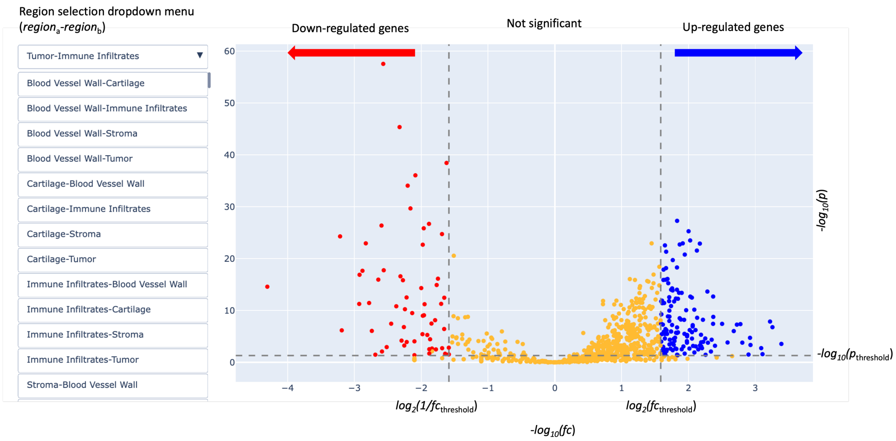


Fig. 13: Visualizing differential gene expression between two regions using a volcano plot. $x$-axis indicates the $\log_2(fc)$ while $y$-axis is $-\log_{10}(p)$. Blue dots are genes that are up-regulated in $region_{\mathrm{A}}$ compared to $region_{\mathrm{B}}$. Red dots are genes that are down-regulated in $region_{\mathrm{A}}$ compared to $region_{\mathrm{B}}$. Yellow dots are genes that have an identical average in the two regions. User can select regions to compare from the drop-down menu where regions are specified in the form "$region_{\mathrm{A}} - region_{\mathrm{B}}$".

## 2. Results

In this section, a detailed analysis of two Visium human lung cancer samples are presented, employing these to demonstrate the capabilities of the SpatialOne pipeline. Furthermore, for the purpose of testing and validation of the pipeline, additional analyses have been executed across a range of publicly available Visium datasets. All the data used in these analysis, including the Visium datasets and the reference sc datasets, has been uploaded to Zenodo: https://zenodo.org/records/12605154. Analysis results are available at https://zenodo.org/records/12628376.

### 2.1. Analysis of Human Lung Cancer Tissue Sections Using SpatialOne

SpatialOne is used to analyze two human lung cancer cancer samples from the publicly available 10x dataset repository. One sample corresponds to a tissue section corresponding to lung neuroendocrine carcinoma (NEC)[1], Figure 14a, and the second sample to a lung

[1] https://www.10xgenomics.com/datasets/human-lung-cancer-11-mm-capture-area-ffpe-2-standard

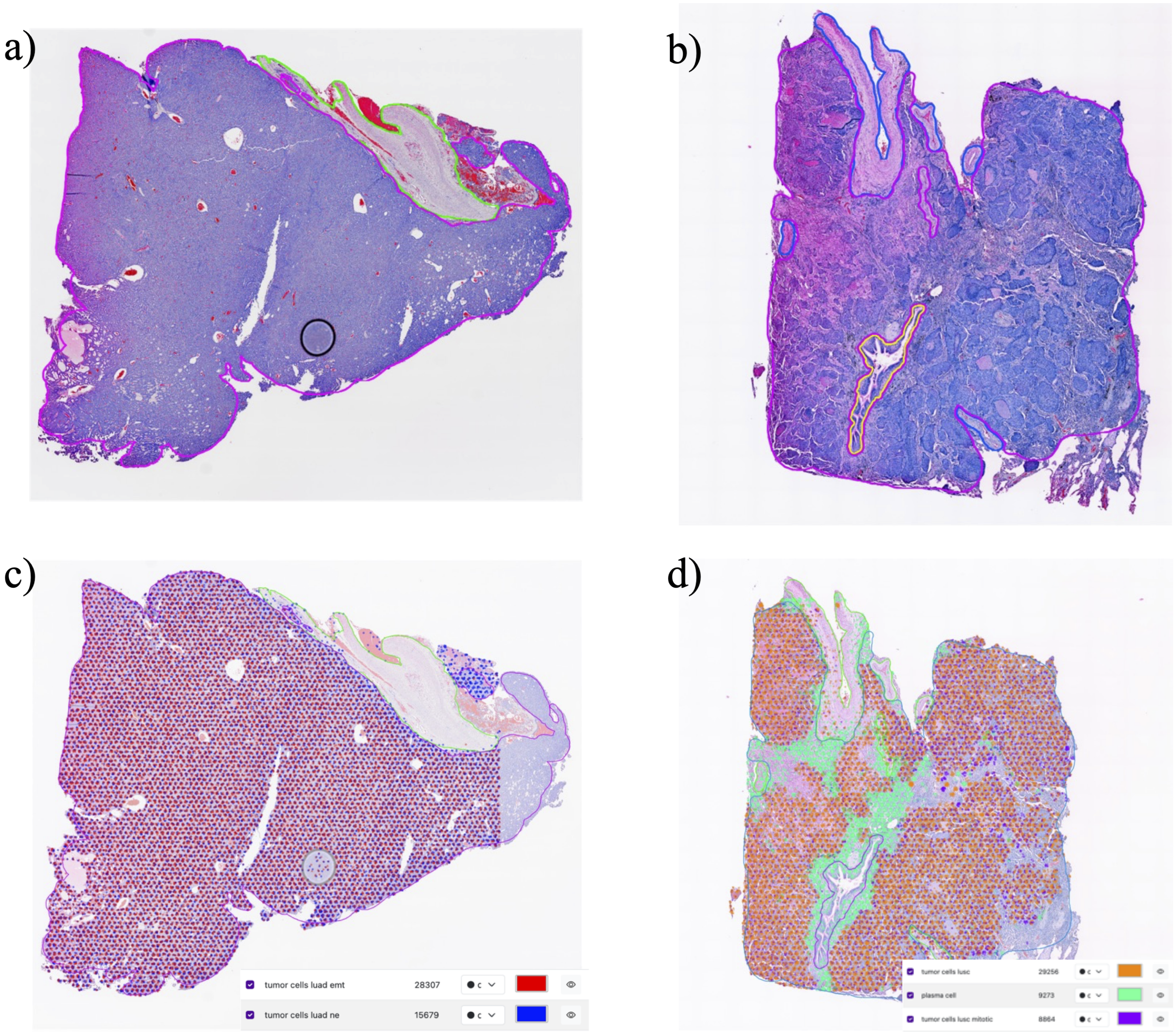


Fig. 14: **a) Lung Neuroendocrine Cancer (NEC) H&E image** with the annotated tumor (pink) and non-tumor (green) regions. **b) Lung Squamous Cell Cancer (SCC) H&E image** with the annotated tumor (pink), blood vessels (blue), and bronchus (yellow). **c) Cell deconvolution output example for NEC:** tumor cells are spread accross the tumor regions but not present in the non-tumor one. **d) Cell deconvolution output example for SCC:** tumor cells are present in the tumor regions; plasma cells concentrate in the border of the tumor regions and around the bronchus.

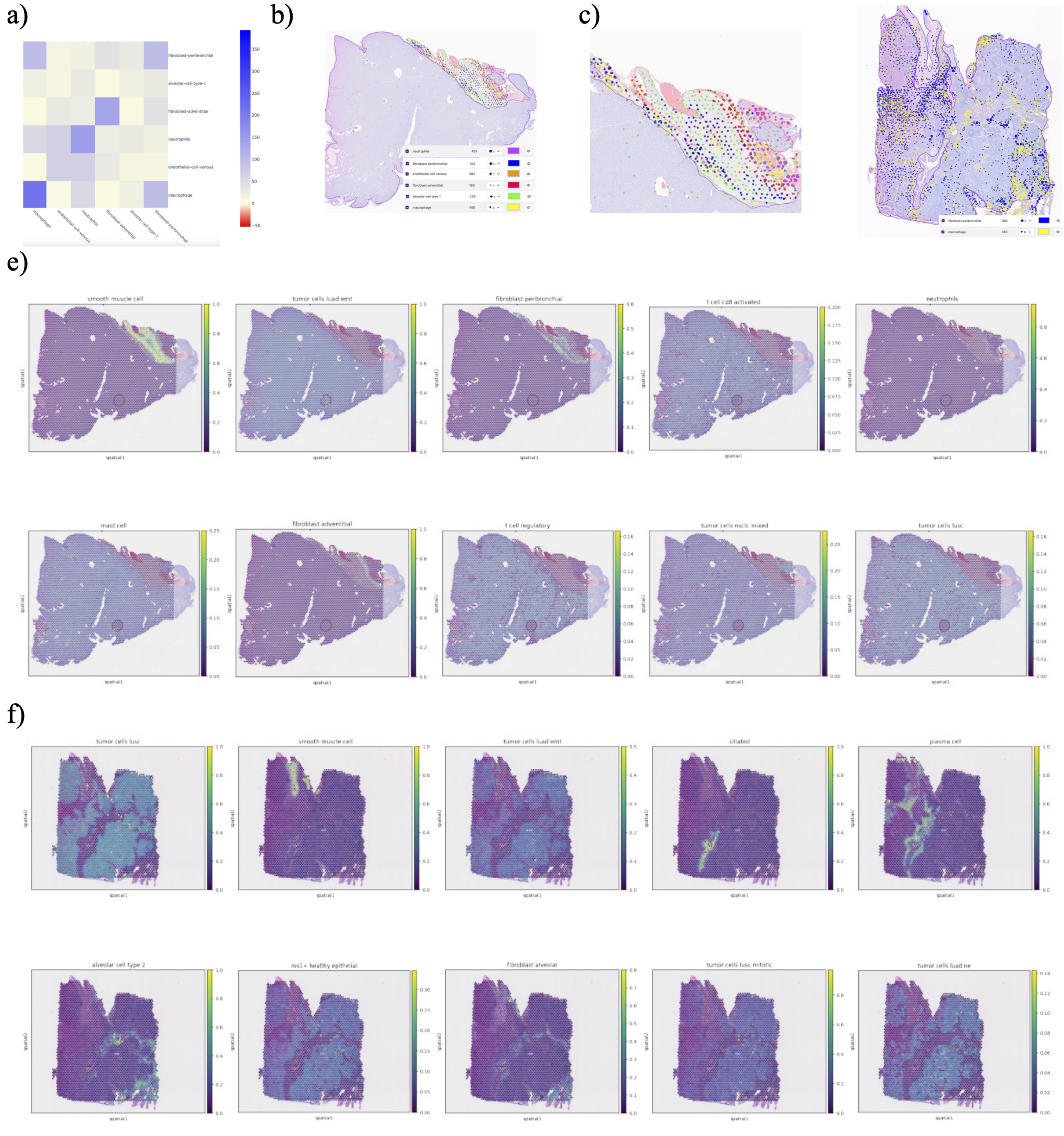


Fig. 15: **a) Detail of the NER clustergram** for the NEC sample. **b & c) Pairs of cells with a high NER Z-score** colocate around the non-tumor area. **d) Macrophages and peribronchial fibroblasts** have a NER Z-score of 61.88 and colocate in the SCC sample. **e & f) Distribution of cells with the highest Moran's I** in the NEC and SCC samples.

tissue section with squamous cell carcinoma (SCC)[2], Figure 14b. Both samples are processed using the Visium Cytassist instrument, using the Visium human transcriptome probe set v2.0, and pre-procssed using SpaceRanger 2.0; the H&E images were acquired using an Olympus VS200 microscope.

[2] https://www.10xgenomics.com/datasets/human-lung-cancer-ffpe-2-standard

CellPose is used as the segmentation algorithm, and Cell2Location uses the Lung Cancer Atlas (LUCA) as the reference single-cell dataset. The LUCA cell atlas is a comprehensive single-cell annotated dataset describing the tumor microenvironment in lung cancer. It integrates data from over a million single cells from patients with different immune subtypes. We filtered the LUCA atlas to contain only single-cell data generated using 10x technology to reduce noise in the data. To validate results, a pathologist annotated regions of interest using only the histological images provided in the dataset.

Supplementary_report_NEC.html andsupplementary_report_SCC.html show the resulting downstream analysis reports of the two samples, while files NeC.tmap and SCC.tmap in supplementary folders N & M allow to visualize the pipeline results on the TissUUmaps spatial viewer Pielawski et al. (2023).

The results section provides an overview of the results obtained through SpatialOne's downstream analysis report

## 2.2. Descriptive Statistics

The "Cell Summary Statistics" table and "Cell Counts" bar plot show that the predominant cell types in the NEC sample are EMT-LUAD tumor cells (28.307), NE-LUAD tumor cells (15.679), and ciliated cells (11.469), while in the SCC sample the most abundant cells are LUSC tumor cells (29.256), plasma cells (9.273), and mitotic LUSC tumor cells (8.864). In both cases, tumor cells concentrate in the tumor areas annotated by the pathologist (Figure 14c). In the SCC sample, LUSC cells are the principal cell type present in the tumor region. Interestingly, plasma cells have a mean of 2.47 cells per spot but a median of 0 cells per spot, indicating that they are highly concentrated in specific spots. This is observed in (Figure 14d) where plasma cells tend to group at the surroundings of the LUSC cell-rich areas.

## 2.3. Spatial Analysis

For the NEC sample, the neighborhood enrichment analysis clustergram indicates that adventitial and peribrochial fibroblast, venous endothelial cells, alveolar cells (type 1) and macrophages form a cluster and tend to be enriched. This is observable in the tissue sample, where those cells tend to concentrate in the non-tumor area and the border of the tumor ((Figure 15a). Focusing on the cell distribution in the sample, we can see that those pairs that seem enriched to the naked eye ((Figure 15c) are also the ones with the highest Z-scores (macrophages-peribronchial fibroblasts: 97.37, venous endothelial cells-neutrophils: 68.44). Similar behavior is observed for the SCC sample (Figure 15d), where we also observe enrichment between macrophages and peribronchial fibroblasts (61.88)

Figures 15e & f show Moran's I for cell auto-correlation in both samples. Cells that present a high Moran's I tend to concentrate in the areas annotated by the pathologist, pointing to a good correspondence between the observable morphological features and the spatial transcriptomics analysis. For the NEC sample, smooth muscle cells and fibroblasts cluster in the non-tumor region, while LUAD tumor cells, CD8+ activated T-cells, and mast cells cluster in the tumor area. For the SCC sample, LUSC and LUAD cells concentrate in the tumor region, and plasma cells surround such clusters.

## 2.4. Comparative Analysis

This section of the HTML report focuses on the differential gene expression analysis of different areas of interest in the provided samples. It allows us to compare both user-pre-annotated regions and spot clusters provided by Space Ranger. Differential expression results can also be checked on diff_exp_annotations.csv and diff_exp_clusters.csv files where the information is presented as tabular data for an easier analysis. To illustrate this functionality we have checked the top 10 upregulated genes in different annotated areas and gene-expression clusters on the tissue and we have consulted their function using the Gene Cards database (Stelzer et al., 2016).

Focussing on the NEC sample, when comparing the tumor with the no-tumor areas (Figure 16a), one can observer that some of the upregulated genes in the tumor areas are linked to Thrombosis (F5, APOH) and immune response and cell signaling (CLEC3A, CXCL13). The upregulated genes in the non-tumor area are related to immunoglobulin generation (IGHA1, IGHG1, IGKC, IGLC3, IGLC2) and to smooth-muscle-related processes (MYH11, ACTA2, MYL9). When comparing cluster regions 7 and 9 (Figure 16b), as region 7 highly overlaps with the annotation for the non-tumor area, we see a similar set of upregulated genes (IGHG1, IGHA1, IGKC, IGLC2, IGLC3, MYH11, ACTA2); nonetheless, when we focus on region 9 - corresponding to the tumor border - we see a different set of upregulated genes with different functions pointing that tumor composition varies across the sample. For instance, we can observe a few genes related to neuroendocrine processes (SGC3, SGC5, and VGF) and neuroendocrine cancer (CGA), as well as genes associated with the creation of protective barriers around tumor cells like MUC3A.

Regarding the SCC sample, Figure 16c presents a volcano plot with the differential gene expression analysis between tumor areas and bronchus ones. It depicts how the tumor area has a significant number of upregulated genes compared to the bronchus one, implying a very different characterization of the two areas. Amongst the most upregulated genes we can identify keratin-related genes (KRT6A) which can act as a cancer biomarker, genes associated with the metabolism of bioactive and toxic substances (AKR1C2, AKR1B10) and with tumorigenesis (SPP1). On the other hand, within the bronchus upregulated genes we can find gene markers for bronchial epithelial cells (TOGARAM2, MUC16, CAPSL, DRC3) but also cancer markers like AKAP14. Regarding the comparison between cluster regions 1 and 8 (Figure 16d), we can observe that cluster 8's upregulated genes are mainly related to immunoglobulin production (IGHM, IGHA1, JCHAIN, IGKC, IGLC3) pointing to it being an area of high immune activity, whilst the ones in cluster 1 involve keratin-related genes (KRT15), genes involved in epithelial cell proliferation and differentiation (PTHLH) and the processing of toxins (CYP4F3). This differentiation within an area annotated as a tumor by a pathologist shows the complexity of the tumor microenvironment and how spatial transcriptomics can help get a better understanding of it.

### 2.5. Validation with additional datasets

Additionally to the in-depth analysis realized with the two lung cancer datasets provided by 10x, we have also tested the SpatialOne pipeline with 3 additional public datasets and with 75 internal Visium samples (not provided).

- Adult Mouse Kidney (FFPE) section[3] obtained from the 10x public repository (pre-processed using SpaceRanger 1.3). The sample has been analyzed using CellPose nuclei model and Cell2location as cell deconvolution (GSE107585 Park et al. (2018) used as reference data). Results available at *adult_mouse_kidney_FFPE_results.zip* supplementary file.
- Adult Mouse Kidney (fresh frozen) coronal section[4] obtained from the 10x public data repository (pre-processed using SpaceRanger 1.1). The sample has been analyzed using CellPose nuclei model and Cell2location as cell deconvolution (GSE107585 Park et al. (2018) used as reference data). Results available at *adult_mouse_kidney_fresh_results.zip* supplementary file.
- Adult Mouse Brain (fresh frozen) section[5] obtained from the 10x public data repository (preprocessed using SpaceRanger 2.0). The sample has been analyzed using CellPOse nuclei model and Cell2Location as cell deconvolution; the single cell dataset provided in the cell2location tutorial[6] has been used as reference dataset (Kleshchevnikov et al., 2020). Results available at *adult_mouse_brain_FFPE_results.zip* supplementary file.

[3] https://www.10xgenomics.com/datasets/adult-mouse-kidney-ffpe-1-standard-1-3-0

[4] https://www.10xgenomics.com/datasets/mouse-kidney-section-coronal-1-standard-1-1-0

[5] https://www.10xgenomics.com/datasets/adult-mouse-brain-coronal-section-fresh-frozen-1-standard

[6] https://cell2location.cog.sanger.ac.uk/tutorial/mouse_brain_snrna/all_cells_20200625.h5ad

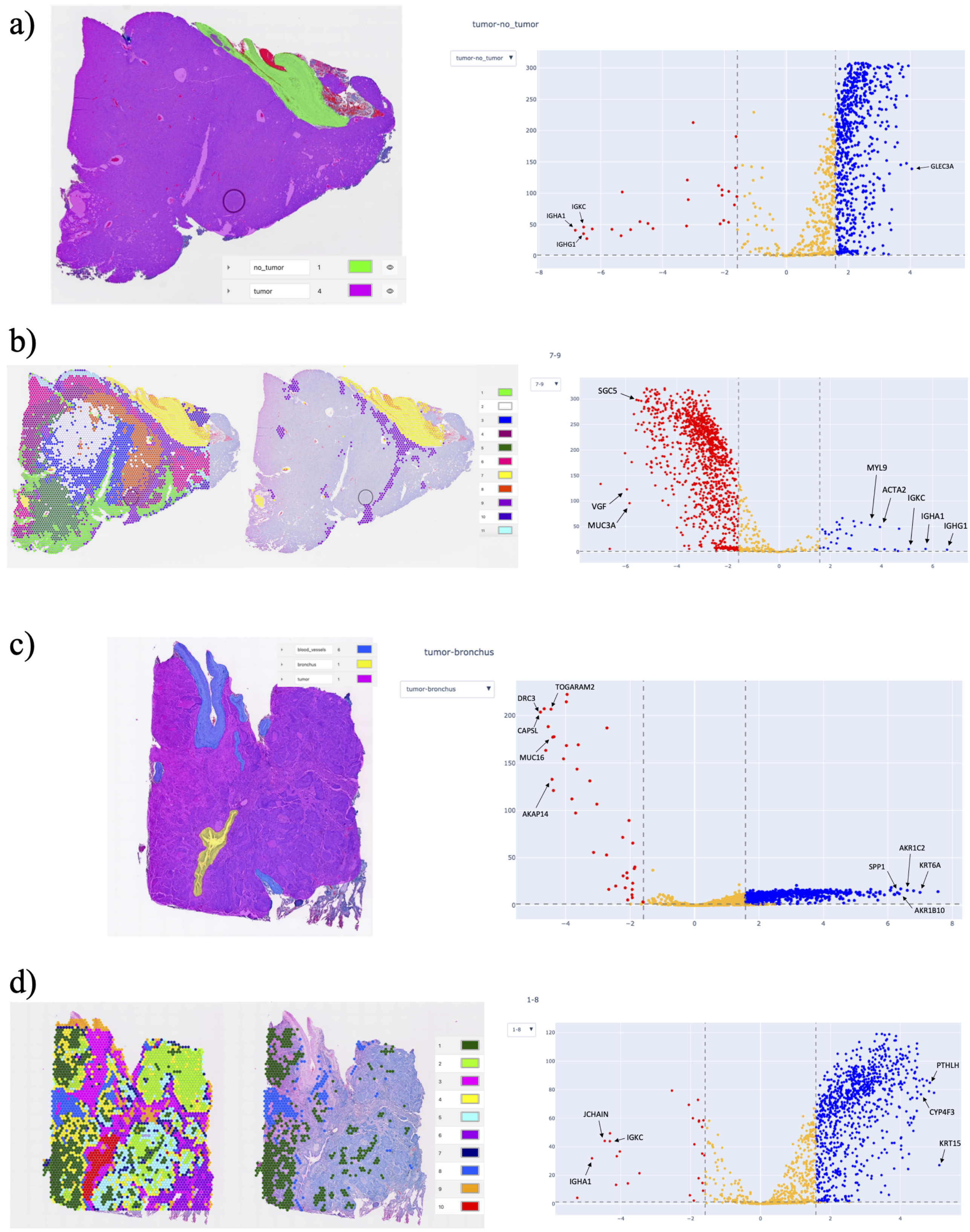


Fig. 16: **a)** Differential gene expression analysis between tumor and non-tumor regions in NEC sample. **b)** Differential gene expression analysis between clusters 7 and 9 in NEC sample. **c)** Differential gene expression analysis between tumour and blood vessel regiosn in SCC sample. **d)** Differential gene expression analysis between clusters 1 and 8 in SCC sample.

## 3. How to Run SpatialOne?

SpatialOne, installation instructions, and examples on how to run it are available on SpatialONE's github repository. For reproducibility and distribution purposes SpatialOne is also provided as a docker container. This section provides details on how to execute SpatialOne from a docker image and run example analysis.

Docker containers are lightweight, portable, and self-sufficient software packages that include everything needed to run a piece of software, including the code, runtime, libraries, and system tools. They encapsulate the application and its dependSencies in a virtual container that can run on any Linux server, regardless of the environment. This isolation ensures that the software runs uniformly and consistently, regardless of where it is deployed. This ensures reproducibility by enabling researchers to share and run identical software setups. By using Docker, SpatialOne can be easily distributed and executed in various computing environments, from local machines to cloud-based infrastructure. This not only facilitates scalability, allowing the pipeline to handle multiple datasets, but also ensures that the results are consistent and reproducible.

SpatialOne container images are provided both for AMD and ARM architectures; its execution requires having docker installed. Compute requirements will depend on the size of the data to be analyzed (resolution of microscopy image, and size of reference single cell dataset) but we recommend running it in a machine with 16 CPU, >120GB RAM, 1 cuda-enabled GPU. SpatialOne can run without a GPU, running cell segmentation (Cellpose) and cell deconvolution (CARD and Cell2Location) in a regular CPU, but this will significantly affect performance and analysis times. Running Hovernet requires a CUDA-enabled gpu. We reccommend running SpatialOne using an cuda-compatible GPU. Some of the libraries used in SpatialOne relies on PyTorch, which does not ensure reproducibility between CPU and GPU executions[7], therefore minor differences should be expected in the cell deconvolution results between CPU and GPU executions.

### 3.1. Set up

For portability and reproducibility SpatialOne is containarized as a docker image. The docker images can be locally built by executing the following commands in a shell terminal. This will download the source code from github and build the docker image.

```
git clone https://github.com/Sanofi-Public/spatialone-pipeline.git
git cd ./spatialone-pipeline
make build
```

Please replace the *Dockerfile* file with *Dockerfile_ARM* in order to compible the docker image for an ARM system.

An AMD-based image is also available in dockerhub:

- https://hub.docker.com/repository/docker/albertpla/spatialone_amd

To directly retrieve the user can use:

```
git clone https://github.com/Sanofi-Public/spatialone-pipeline.git
cd spatialone-pipeline
docker pull albertpla/spatialone_amd:latest
docker tag albertpla/spatialone_amd:latest spatialone-pipeline:latest
```

To run the pipeline use one of the following commands depending on the availability of a CUDA-enabled GPU:

- GPU-based:

```
docker run --gpus device=${GPU_DEVICE_ID} -it -v ${HOST_DATA_PATH}:/app/data -it spatialone-pipeline
```

- CPU-based:

```
docker run -it -v ${HOST_DATA_PATH}:/app/data -it spatialone-pipeline
```

We recommend setting up the following system variables:

- **HOST_DATA_PATH** defines the path the SpatialOne pipeline will read the data from

```
export $HOST_DATA_PATH = /Users/user_name/Documents/SpatialOnePipeline
```

- **GPU_DEVICE_ID** defines the id of the GPU that will run the analyses

```
export $GPU_DEVICE_ID=0
```

Please refer to the project https://github.com/Sanofi-Public/spatialone-pipeline for more detailed instructions.

[7] https://pytorch.org/docs/stable/notes/randomness.html

## 3.2. Data Preparation

SpatialOne takes its data from the folder specified in **HOST_DATA_PATH**, which must respect the following structure:

```
HOST_DATA_PATH/
   conf/
      visium_config_flow.yaml
   prep/
      sample1/
         #input files
      sample2/
         #input files
   reference/
      # input reference data
      reference_cell_atlas.h5ad
      ...
   results/
      #populated by the pipeline
```

- **conf** contains the YAML configuration file that will define the pipeline setup. It must respect the following name: visium_config_flow.yaml
- **prep** contains the different experiment data. Each folder corresponds to a different experiment which must contain the SpaceRanger outputs defined in Section 3.2.1
- **reference** contains the single cell datasets that will be used as reference for the deconvolution. These must be anndata objects following the formatting defiend in 3.2.2. It follows this naming convention *datasetName*_cell_atlas.h5ad.
- **results** will contain the outputs of the analyiss. Each analysis will generate an output folder with a name matching the experiment one.

### 3.2.1. Input files

SpatialOne requires the following files, which can be obtained from SpaceRanger's outputs. These files must be stored in the *prep* folder within a subfolder named after the experiment. They must adhere to the following naming convention:

- **raw_feature_bc_matrix.h5**
- **scalefactors_json.json**
- **tissue_hires_image.png**
- **tissue_lowres_image.png**
- **tissue_positions_list.csv**
- **wsi.tif**: High resolution image obtained from a mycrocospe. Its resolution must be aligned to the *scalefactors_json.json* file.
- **gene_graph_clusters.csv**: A csv containing the clustering results from the space ranger analysis. We reccomend using the output of the graph-based clustering, but it also works with the results of K-means clustering analysis provided by SpaceRanger.
- **parameters.csv** A csv containing basic metadata on the experiment properties.
- **annoations.geojson** (optional): A geojson file defining regions of interest that will be analyzed by the pipeline.
- **web_summary.html** (optional): The SpaceRanger resulting html report. If it is provided, it will be embeded in the SpatialOne output report.

### 3.2.2. Reference datasets

Reference single cell datasets must be stored under the *reference* folder and using the *cell_atlas.h5ad* suffix.

In order to be readable both to CARD and Cell2Location single cell reference datasets must contain the gene expression profiles for the different cell types to be used in the deconvolution step. They must be formatted as a basic anndata object [8] with the following specific requirements: *1 - anndata.X sparse table should contain raw gene counts, where the rows correspond to the cell types and the columns to the genes. 2 - anndata.obs dataframe shoud contain at least two columns with batch and cell types labels (default colnames used at configuration file will be set to "batch" and "cell_type" respectively) 3 - anndata.var_names should align to the gene names generated by cell Ranger. By the default gene symbols should be used.*

## 3.3. SpatialOne Outputs

### 3.3.1. Output Files

This list describes the outputfiles generated by SpatialOne. Depending on the analysis configuration, some of these files may not be present.

- `cell2location_results.csv` / `card_results.csv`: CSV file containing the estimated cell counts per spot.

[8] https://anndata.readthedocs.io/en/latest/

- `cellpose_cell_segmentation_crop.npy` / `hovernet_cell_segmentation_crop`: numpy array containing the masks of all segmented cells.
- `cellpose_cell_segmentation_full.npy` / `hovernet_cell_segmentation_full.npy`: numpy array containing the masks of all segmented cells.
- `cells_adata.h5`: Anndata object containing all cell related information (location, estimated cell type, barcode, etc.)
- `cells_df.csv`: CSV file containing the polygons defining the segmented cells, their center, and the spot they belong to (if any).
- `cells_layer.png`: PNG image with the contours of the segmented cells.
- `experiment_tmap.tmap`: TissUUmaps configuration file for results visualization.
- `gene_qc.csv`: CSV file containing computed quality control metrics for each gene present in the original sample.
- `merged_cells_df.csv`: CSV file containing cell information: location, spot it belongs to (if in spot), morphological cluster it belongs to (if any), and estimated cell type (if in spot)
- `merged_spots_df.csv`: CSV file containing spot information: location, associated barcode, number of cells identified in the spot, qc metrics, counts for each cell type identified in the deconvolution, gene expression levels for the genes specified in the data merge step
- `morphological_clusters.csv`: CSV file containing cell morphological features and results of cell morphological cluster step.
- `overall_qc.csv`: Aggregated quality control metrics for the whole sample
- `piechart_df.csv`: CSV file containing cell proportions at each spot. Note that the cell proportions are obtained from the estimated cell counts and not from the raw deconvolution results.
- `reports`: Folder containing the spatial analysis HTML reports. `report.html` corresponds to the whole slide report whilst `regionX_report.html` correspond to annotation-specific regions.
- `run_configs.json`: summary of the analysis configuration
- `spot_qc.csv`: CSV file containing computed quality control metrics for each spot
- `spots_adata.h5`: Anndata object containing relevant spot information (location, cell counts, deconvolution results, gene expression, clustering results, qc results)
- `spots_df.csv`: CSV file containing the spot locations and the number of cells in each one.
- `spots_layer.png`: PNG image containing the spot locations for visualization purposes.

#### 3.3.2. Visualizing Outputs in TissUUmaps

To visualize SpatialOne outputs in TissUUmaps, follow these instructions:

1. Install TissUUmaps following its official instructions[9]
2. Run TissUUmaps
3. Load the .tmaps file saved at *data/results/experiment_name/experiment_tmap.tmap*

### 3.4. Demonstration

To obtain the required data to replicate the analysis presented in this experiment run the following script:

```
./download_experiment_data.sh
```

To replicate the analyses presented in this paper checkout the code repository from GitHub and execute the *run instructions algorithm*. GPU execution:

```
git clone https://github.com/Sanofi-Public/spatialone-pipeline.git
cd spatialone-pipeline
./run_instructions_SO.sh --gpu
```

CPU execution:

```
git clone https://github.com/Sanofi-Public/spatialone-pipeline.git
cd spatialone-pipeline
./run_instructions_SO.sh --cpu
```

This will will download the project from github, build an AMD-based docker image, download the input experiment data, and run the analyses. Please adapt the script accordingly if you plan to run the analyses using the ARM-based docker image by building the docker image using the *Dockerfile_arm* instead.

Note that these examples require a machine with approximately 200GB of memory. If the user is accessing the internet through a proxy, the makefile and/or the .env files allow to configure proxy access.

### 3.5. Hardware compatibility

SpatialOne has been tested in the following systems:

- m5.12xlarge AWS EC2 instance with Amazon Linux 2 (Kernel-5.4)

[9] https://tissuumaps.github.io/installation/

- p3.8xlarge AWS EC2 GPU instance with Amazon Linux 2 (Kernel-5.4)
- Ubuntu 22.4 Linux Machine with a GPU
- Apple M2 Pro 16 GB Ventura.

Note that for Apple Silicon-based chips, such as the M1 and M2 processors, the docker-ARM image needs to be used. Tests in the Ubuntu and Apple M2 machines were realized with smaller datasets due to its memory limitations.

## 4. Comparison With Other Methods

The spatial transcriptomics analysis landscape is composed of tools covering different analysis aras and directed to different users. Table 5 describes a variety of existing methods and compares their capabilities with the ones of SpatialOne. Tools like ST Tools and Spacemake are designed to analyze raw spatial transcriptomic data, focussing on aligning the data with a reference geneome and establishing the spatial gene counts, they also perform basic analysis at the provided Visium resolution, providing comprehensive basic analyses from the ground up. They are similar to 10X SpaceRanger, the official 10x Visium data processing pipeline, but they expand their capabilities to other spatial trancriptomics technologies. Other tools, such as Squidpy and Giotto, offer a suite of functionalities to study spatial gene expression patterns, clustering, and visualization. Seurat, originally developed for single-cell RNA sequencing data, has since been adapted to work with spatial data, expanding its utility. Here we will discuss these comparisons in greater detail.

It is important to note that SpatialOne leverages outputs from SpaceRanger. This choice allows SpatailOne to delegate the processing of raw data to a tool validated by the technology manufacturer. While tools such as Spacemake and STTools can also process raw Vsium files, SpaceRanger provides comparable results. Furthermore, since the release of both Spacemake and STTools, SpaceRanger has kept up to date with the changes to the Visium platform and has consistently added new analysis capabilities, with the latest Visium updates covering most, if not all, of the capabilities of the two methods in question. For these reasons, SpatialOne was designed to integrate seamlessly with SpaceRanger outputs, maintaining efficiency and avoiding the constant adaptation of basic analysis capabilities as the Visium platform evolves.

In addition to its compatibility with SpaceRanger, SpatialOne also incorporates imaging data, which facilitates complex downstream analysis at an estimated single-cell resolution. Most existing pipelines consider all information within a spot as a singular data point, which limits the ability to conduct analyses such as cell-cell neighborhood enrichment tests, infiltration analysis, and co-occurrence patterns. These analyses have been traditionally reserved for sub-cellular resolution technologies such as Xenium or CosMx. For instance, SRT-Server (Yang and Zhou, 2024), a recent end-to-end pipeline, relies solely on expression data, limiting its capacity for genuine spatial single-cell level analysis. By incorporating imaging data, SpatialOne also facilitates sub-domain analysis, allowing for comparisons between different regions on the tissue through annotation files.

The platform is designed to simplify working with spatial transcriptomic data for users of varying expertise levels. For advanced users familiar with the packages involved in the analysis, SpatialOne platform provides adjustable parameters to tailor the analysis of the user. On the other hand, wet lab scientists, who may posess deep biological understanding but limited computational skills, can utilize the default set of parameters to run their analyses efficiently. While more advanced users may be able to create detailed pipelines on their own, this platform provides a way to streamline the nalaysis work in a scalable, stable, and reliable framework, eliminating the need for the tedious processing and dependency management, typical of large-scale engineering efforts. In addition, SpatialOne provides stable visualizations and reporting options that facilitate the communication and display of results, such as through HTML reports or TissUUmaps visualizations, which can otherwise be cumbersome.

| Features | | SpatialOne | Giotto | Seurat | Squidpy | STUtility | Semla | PathML | Spacemake | ST Tools | ST Pipeline | Space Ranger | SRT-Server |
|---|---|---|---|---|---|---|---|---|---|---|---|---|---|
| Framework | Programming language | R/Python | R/Python | R | Python | R | R | Python | Python | R/Python | Python | Bash/Python | R/Python |
| | Data Object | Anndata | Giotto | Seurat | anndata | Seurat | Seurat | Slide | Anndata | Seurat | - | - | Seurat |
| | End-to-End Execution | ✓ | ✗ | ✗ | ✗ | ✗ | ✗ | ✗ | ✓ | ✓ | ✓ | ✓ | ✓ |
| | Containerized Deployment | ✓ | ✓ | ✗ | ✗ | ✗ | ✗ | ✗ | ✗ | ✗ | ✗ | ✓? | ✓ |
| Spatial Transcriptomics Analysis | Quality Check | ✓ | ✓ | ✓ | ✓ | ✗ | ✗ | ✗ | ✓ | ✓ | ✗ | ✓ | ✓ |
| | Dimensionality Reduction | ✓* | ✓ | ✓ | ✓ | ✓ | ✗ | ✗ | ✓ | ✓ | ✗ | ✓ | ✓ |
| | Differential Gene Expression | ✓ | ✓ | ✓ | ✓ | ✓ | ✓ | ✗ | ✓ | ✓ | ✗ | ✓ | ✓ |
| | Gene Expression Clustering | ✓ | ✓ | ✓ | ✓ | ✓ | ✓ | ✗ | ✓ | ✓ | ✗ | ✓ | ✓ |
| | Spot level Statistical Analysis | ✓ | ✓ | ✓ | ✓ | ✗ | ✓ | ✗ | ✓ | ✓ | ✗ | ✓ | ✓ |
| | Spot Cell type Deconvolution | ✓ | ✓ | ✓ | ✓ | ✗ | ✓ | ✗ | ✓ | ✗ | ✗ | ✓ | ✓ |
| Spatial Image Analysis | Image Feature Extraction | ✓ | ✗ | ✗ | ✓ | ✗ | ✗ | ✓ | ✗ | ✗ | ✗ | ✗ | ✗ |
| | Spot Level Morphological Clustering | ✗ | ✗ | ✗ | ✓ | ✗ | ✗ | ✓ | ✗ | ✗ | ✗ | ✗ | ✗ |
| | Cell Segmentation | ✓ | ✗ | ✗ | ✓ | ✗ | ✗ | ✓ | ✗ | ✗ | ✗ | ✗ | ✗ |
| Transcriptomics & Image integration | Spatial Gene Expression visualization | ✓ | ✓ | ✓ | ✓ | ✓ | ✓ | ✗ | ✓ | ✓ | ✗ | ✗ | ✓ |
| | Single-cell type estimation | ✓ | ✗ | ✗ | ✗ | ✗ | ✗ | ✗ | ✗ | ✗ | ✗ | ✗ | ✗ |
| | Cell Level Morphological Clustering | ✓ | ✗ | ✗ | ✗ | ✗ | ✗ | ✓ | ✗ | ✗ | ✗ | ✗ | ✗ |
| | Cell level Statistical Analysis | ✓ | ✗ | ✗ | ✗ | ✗ | ✗ | ✗ | ✗ | ✗ | ✗ | ✗ | ✗ |
| Supported technology | Sequencing-based ST | ✓ | ✓ | ✓ | ✓ | ✓ | ✓ | ✓** | ✓ | ✓ | ✓ | ✓ | ✓ |
| | Imaging-based ST | ✗ | ✓ | ✓ | ✓ | ✗ | ✗ | ✓** | ✗ | ✗ | ✗ | ✗ | ✓ |
| Preprocessing | RNAseq and barcoding alignment | ✗ | ✗ | ✗ | ✗ | ✗ | ✗ | ✗ | ✓ | ✓ | ✓ | ✓ | ✗ |
| | Gene count matrix generation | ✗ | ✗ | ✗ | ✗ | ✗ | ✗ | ✗ | ✓ | ✓ | ✓ | ✓ | ✗ |
| E2E Reporting | Whole sample report | ✓ | ✗ | ✗ | ✗ | ✗ | ✗ | ✗ | ✓ | ✓ | ✓ | ✓ | ✓ |
| | Region level report | ✓ | ✗ | ✗ | ✗ | ✗ | ✗ | ✗ | ✗ | ✗ | ✗ | ✗ | ✗ |
| | Output files of each pipeline step | ✓ | ✗ | ✗ | ✗ | ✗ | ✗ | ✗ | ✓ | ✓ | ✓ | ✓ | ✓ |

**Table 5.** Comparison of spatial transcriptomics analyis methods. *Relies on Space Ranger inputs. **Can work with any technology providing high-resolution images.

## References

F. Avila Cobos, J. Alquicira-Hernandez, J. E. Powell, P. Mestdagh, and K. De Preter. Benchmarking of cell type deconvolution pipelines for transcriptomics data. *Nature communications*, 11(1):5650, 2020.

Y. Benjamini and Y. Hochberg. Controlling the false discovery rate: a practical and powerful approach to multiple testing. *Journal of the Royal statistical society: series B (Methodological)*, 57(1):289–300, 1995.

T. Biancalani, G. Scalia, L. Buffoni, R. Avasthi, Z. Lu, A. Sanger, N. Tokcan, C. R. Vanderburg, Å. Segerstolpe, M. Zhang, et al. Deep learning and alignment of spatially resolved single-cell transcriptomes with tangram. *Nature methods*, 18(11):1352–1362, 2021.

A. Dekkers and E. Aarts. Global optimization and simulated annealing. *Mathematical programming*, 50:367–393, 1991.

E. Drokhlyansky, C. S. Smillie, N. Van Wittenberghe, M. Ericsson, G. K. Griffin, G. Eraslan, D. Dionne, M. S. Cuoco, M. N. Goder-Reiser, T. Sharova, O. Kuksenko, A. J. Aguirre, G. M. Boland, D. Graham, O. Rozenblatt-Rosen, R. J. Xavier, and A. Regev. The human and mouse enteric nervous system at single-cell resolution. *Cell*, 182(6):1606–1622.e23, 2020. Publisher: Elsevier.

J. Du, Y.-C. Yang, Z.-J. An, M.-H. Zhang, X.-H. Fu, Z.-F. Huang, Y. Yuan, and J. Hou. Advances in spatial transcriptomics and related data analysis strategies. *Journal of Translational Medicine*, 21(1):1–21, 2023.

N. Ganganath, C.-T. Cheng, and K. T. Chi. Data clustering with cluster size constraints using a modified k-means algorithm. In *2014 International Conference on Cyber-Enabled Distributed Computing and Knowledge Discovery*, pages 158–161. IEEE, 2014.

S. Graham, Q. D. Vu, S. E. A. Raza, A. Azam, Y. W. Tsang, J. T. Kwak, and N. Rajpoot. Hover-net: Simultaneous segmentation and classification of nuclei in multi-tissue histology images. *Medical image analysis*, 58:101563, 2019. Publisher: Elsevier.

S. Graham, M. Jahanifar, A. Azam, M. Nimir, Y. W. Tsang, K. Dodd, E. Hero, H. Sahota, A. Tank, K. Benes, N. Wahab, F. Minhas, S. E. A. Raza, H. El Daly, K. Gopalakrishnan, D. Snead, and N. Rajpoot. Lizard: A large-scale dataset for colonic nuclear instance segmentation and classification. *CoRR*, abs/2108.11195:684–693, 2021.

W. Hendriksation. Frigidum, 2020. URL https://pypi.org/project/frigidum/.

V. Kleshchevnikov, A. Shmatko, E. Dann, A. Aivazidis, H. W. King, T. Li, A. Lomakin, V. Kedlian, M. S. Jain, J. S. Park, et al. Comprehensive mapping of tissue cell architecture via integrated single cell and spatial transcriptomics. *BioRxiv*, pages 2020–11, 2020.

V. Kleshchevnikov, A. Shmatko, E. Dann, A. Aivazidis, H. W. King, T. Li, R. Elmentaite, A. Lomakin, V. Kedlian, A. Gayoso, and others. Cell2location maps fine-grained cell types in spatial transcriptomics. *Nature biotechnology*, 40(5):661–671, 2022. Publisher: Nature Publishing Group US New York.

M. Y. Lee, J. S. Bedia, and S. S. Bhate. Cellseg: a robust, pre-trained nucleus segmentation and pixel quantification software for highly multiplexed fluorescence images. *BMC Bioinformatics*, pages 23–46, 2022. Publisher: Springer.

B. Li, W. Zhang, C. Guo, H. Xu, L. Li, M. Fang, Y. Hu, X. Zhang, X. Yao, M. Tang, et al. Benchmarking spatial and single-cell transcriptomics integration methods for transcript distribution prediction and cell type deconvolution. *Nature methods*, 19(6):662–670, 2022.

H. Li, J. Zhou, Z. Li, S. Chen, X. Liao, B. Zhang, R. Zhang, Y. Wang, S. Sun, and X. Gao. A comprehensive benchmarking with practical guidelines for cellular deconvolution of spatial transcriptomics. *Nature Communications*, 14(1):1548, 2023.

B. Liu, Y. Li, and L. Zhang. Analysis and visualization of spatial transcriptomic data. *Frontiers in Genetics*, 12:785290, 2022.

M. D. Luecken and F. J. Theis. Current best practices in single-cell rna-seq analysis: a tutorial. *Molecular systems biology*, 15(6):e8746, 2019.

Y. Ma and X. Zhou. Spatially informed cell-type deconvolution for spatial transcriptomics. *Nature biotechnology*, 40(9):1349–1359, 2022. Publisher: Nature Publishing Group US New York.

P. A. Moran. Notes on continuous stochastic phenomena. *Biometrika*, 37(1/2):17–23, 1950.

M. Pachitariu and C. Stringer. Cellpose 2.0: how to train your own model. *Nature methods*, 19(12):1634–1641, 2022. Publisher: Nature Publishing Group US New York.

G. Palla, H. Spitzer, M. Klein, D. Fischer, A. C. Schaar, L. B. Kuemmerle, S. Rybakov, I. L. Ibarra, O. Holmberg, I. Virshup, and others. Squidpy: a scalable framework for spatial omics analysis. *Nature methods*, 19(2):171–178, 2022a. Publisher: Nature Publishing Group US New York.

G. Palla, H. Spitzer, M. Klein, D. Fischer, A. C. Schaar, L. B. Kuemmerle, S. Rybakov, I. L. Ibarra, O. Holmberg, I. Virshup, et al. Squidpy: a scalable framework for spatial omics analysis. *Nature methods*, 19(2):171–178, 2022b.

J. Park, R. Shrestha, C. Qiu, A. Kondo, S. Huang, M. Werth, M. Li, J. Barasch, and K. Suszták. Single-cell transcriptomics of the mouse kidney reveals potential cellular targets of kidney disease. *Science*, 360(6390):758–763, 2018. Publisher: American Association for the Advancement of Science.

F. Pedregosa, G. Varoquaux, A. Gramfort, V. Michel, B. Thirion, O. Grisel, M. Blondel, P. Prettenhofer, R. Weiss, V. Dubourg, J. Vanderplas, A. Passos, D. Cournapeau, M. Brucher, M. Perrot, and E. Duchesnay. Scikit-learn: Machine learning in Python. *Journal of Machine Learning Research*, 12:2825–2830, 2011.

N. Pielawski, A. Andersson, C. Avenel, A. Behanova, E. Chelebian, A. Klemm, F. Nysjö, L. Solorzano, and C. Wählby. TissUUmaps 3: Improvements in interactive visualization, exploration, and quality assessment of large-scale spatial omics data. *Heliyon*, 9(5), 2023. Publisher: Elsevier.

Y. Rubner, C. Tomasi, and L. Guibas. A metric for distributions with applications to image databases. In *Sixth International Conference on Computer Vision (IEEE Cat. No.98CH36271)*, pages 59–66, 1998. doi: 10.1109/ICCV.1998.710701.

S. Salcher, G. Sturm, L. Horvath, G. Untergasser, G. Fotakis, E. Panizzolo, A. Martowicz, G. Pall, G. Gamerith, M. Sykora, F. Augustin, K. Schmitz, F. Finotello, D. Rieder, S. Sopper, D. Wolf, A. Pircher, and Z. Trajanoski. High-resolution single-cell atlas reveals diversity and plasticity of tissue-resident neutrophils in non-small cell lung cancer. *bioRxiv*, 2022. doi: 10.1101/2022.05.09.491204. URL https://www.biorxiv.org/content/early/2022/05/10/2022.05.09.491204.

D. Schapiro, H. W. Jackson, S. Raghuraman, J. R. Fischer, V. R. Zanotelli, D. Schulz, C. Giesen, R. Catena, Z. Varga, and B. Bodenmiller. histocat: analysis of cell phenotypes and interactions in multiplex image cytometry data. *Nature methods*, 14(9):873–876, 2017.

V. Singhal, N. Chou, J. Lee, J. Liu, W. K. Chock, L. Lin, Y.-C. Chang, E. Teo, H. K. Lee, K. H. Chen, and S. Prabhakar. Banksy: A spatial omics algorithm that unifies cell type clustering and tissue domain segmentation. *Nature Genetics*, 56(4):431–441, 2024. Publisher: Nature Publishing Group.

G. Stelzer, N. Rosen, I. Plaschkes, S. Zimmerman, M. Twik, S. Fishilevich, T. I. Stein, R. Nudel, I. Lieder, Y. Mazor, et al. The genecards suite: from gene data mining to disease genome sequence analyses. *Current protocols in bioinformatics*, 54(1):1–30, 2016.

V. Svensson, S. Teichmann, and O. Stegle. Spatialde: identification of spatially variable genes. *Nature Methods*, 15(4):343–346, 2018. Publisher: Nature Publishing Group.

L. Tosti, Y. Hang, O. Debnath, S. Tiesmeyer, T. Trefzer, K. Steiger, F. W. Ten, S. Lukassen, S. Ballke, A. A. Kühl, et al. Single-nucleus and in situ rna–sequencing reveal cell topographies in the human pancreas. *Gastroenterology*, 160(4):1330–1344, 2021.

M. R. Vahid, E. L. Brown, C. B. Steen, W. Zhang, H. S. Jeon, M. Kang, A. J. Gentles, and A. M. Newman. High-resolution alignment of single-cell and spatial transcriptomes with CytoSPACE. *Nature Biotechnology*, pages 1–6, 2023. Publisher: Nature Publishing Group US New York.

S. van der Walt, J. L. Schönberger, J. Nunez-Iglesias, F. Boulogne, J. D. Warner, N. Yager, E. Gouillart, T. Yu, and the scikit-image contributors. scikit-image: image processing in Python. *PeerJ*, 2:e453, 6 2014. ISSN 2167-8359. doi: 10.7717/peerj.453. URL https://doi.org/10.7717/peerj.453.

S. Yang and X. Zhou. Srt-server: powering the analysis of spatial transcriptomic data. *Genome Medicine*, 16(1):18, 2024.

Y. Zhang, X. Lin, Z. Yao, D. Sun, X. Lin, X. Wang, C. Yang, and J. Song. Deconvolution algorithms for inference of the cell-type composition of the spatial transcriptome. *Computational and Structural Biotechnology Journal*, 21:176–184, 2023.

Q. Zhou, X. Su, G. Jing, S. Chen, and K. Ning. Rna-qc-chain: comprehensive and fast quality control for rna-seq data. *BMC genomics*, 19(1): 1–10, 2018.
